# ZDHHC-Mediated Protein S-Palmitoylation in Cancer: Epigenetic Interfaces, Structural Logic and Therapeutic Targeting

**DOI:** 10.7150/ijms.130609

**Published:** 2026-07-13

**Authors:** Xinyi Chen, Duo Xu, Yongbiao Huang, Xianglin Yuan

**Affiliations:** Department of Oncology, Tongji Hospital, Tongji Medical College, Huazhong University of Science and Technology, Wuhan, Hubei 430030, P.R. China.

**Keywords:** S-palmitoylation, ZDHHC palmitoyltransferases, depalmitoylases, oncogenic signaling, cancer metabolism, tumor immunity

## Abstract

Protein S-palmitoylation, the reversible thioesterification of cysteine side chains, is emerging as a druggable post-translational modification that couples membrane topology to oncogenic, metabolic, immune, and epigenetic networks in cancer. ZDHHC palmitoyltransferases and depalmitoylating enzymes, including acyl-protein thioesterases and palmitoyl-protein thioesterase 1, constitute a dynamic circuitry that governs the localization, stability, and signaling competence of key regulators of tumor growth, metabolic adaptation, and immune phenotype. Here, we synthesize recent structural and chemical biology advances that clarify how human ZDHHC enzymes achieve acyl-chain recognition and substrate engagement. Structural studies show that these enzymes adopt a four-transmembrane, “tent-like” fold, in which the helices create a membrane-embedded cavity for acyl-chain accommodation. We also discuss how ankyrin-repeat domains and accessory partners shape substrate recruitment and subcellular localization, and we highlight emerging high-throughput platforms that enable quantitative profiling of isoform- and site-selective modulators. We then discuss how ZDHHC-substrate circuits rewire canonical growth-factor signaling and epithelial-mesenchymal transition programs, metabolic and ferroptotic control nodes, innate immune sensing, and chromatin-linked regulation. These convergent mechanisms position ZDHHC-mediated S-palmitoylation as a context-dependent regulator of tumor progression, therapy response, ferroptosis sensitivity, and immune phenotype. Finally, we outline a translational framework encompassing clinical-stage PPT1 inhibitors, selective ABHD17 blockade, emerging ZDHHC modulators, substrate-competitive strategies targeting checkpoint palmitoylation, and selected comparator approaches affecting Wnt and Hedgehog ligand lipidation. Current evidence positions ZDHHC-mediated S-palmitoylation as a regulatory layer with potential biomarker and therapeutic relevance; however, not all reported ZDHHC-substrate associations carry equivalent evidentiary weight. Mechanisms supported by convergent site-directed, genetic, biochemical, functional, and in vivo evidence should be distinguished from associations inferred mainly from expression profiling, overexpression systems, single-model observations, or broad pharmacological perturbation. Clinical translation remains preliminary and is constrained by isoform selectivity, substrate redundancy, incomplete pharmacodynamic read-outs, and the absence of validated biomarker-guided patient stratification.

## 1. Introduction

Protein S-palmitoylation is a dynamic and reversible lipid modification in which a 16-carbon palmitate moiety is covalently attached to specific cysteine residues of target proteins via a thioester bond 1. This reaction increases protein hydrophobicity and thereby regulates membrane anchoring, subcellular trafficking, protein stability and protein-protein interactions 1. In mammalian cells, S-palmitoylation is catalyzed mainly by palmitoyltransferases that contain a conserved Asp-His-His-Cys (DHHC) motif—collectively known as the ZDHHC family—whereas removal of the acyl group is mediated by thioesterases such as acyl-protein thioesterases 1/2 (APT1/2), palmitoyl-protein thioesterases 1/2 (PPT1/2) and members of the α/β-hydrolase domain-containing 17 (ABHD17) family 2. Together, these enzymes form a dynamic palmitoylation-depalmitoylation cycle that maintains the cellular balance of S-acylation and fine-tunes the residency and signaling competence of peripheral membrane proteins 3. Accumulating evidence indicates that this cycle regulates multiple tumor-associated processes, including proliferation, migration, invasion, immune evasion, metabolic reprogramming, and the maintenance of cancer stem-like states, and can stabilize both oncogenic drivers and selected tumor suppressors 4, 5. Beyond these phenotypic effects, emerging studies further suggest that S-palmitoylation also interfaces with chromatin-associated factors and DNA-damage responses, thereby extending its influence from membrane signaling to epigenetic regulation.

S-palmitoylation serves as a key regulatory mechanism across several oncogenic signaling cascades. In canonical pathways such as EGFR, Hedgehog, and Wnt, palmitoylation influences membrane anchoring and the conformational activation of signaling proteins 6, 7. Protein palmitoylation can be classified into three principal forms according to the chemistry and site of lipid attachment: S-palmitoylation on cysteine residues 8, N-palmitoylation on amino groups 9, and O-acylation on serine residues 10. In cancer, dysregulated expression of ZDHHC family members and of other acyltransferases, including HHAT, which mediates Hedgehog N-palmitoylation 9, and PORCN, which catalyzes Wnt O-acylation 10, has been documented across multiple tumor types, with context-dependent oncogenic or tumor-suppressive effects 1; however, these non-ZDHHC enzymes are discussed here only as conceptual comparators and upstream ligand-modifying systems, whereas the principal focus of this review remains ZDHHC-mediated S-palmitoylation in cancer. Targeting these lipid-modifying enzymes has therefore emerged as a promising therapeutic strategy. Pharmacologic inhibition of palmitoyltransferases such as DHHC20, HHAT, or PORCN can enhance the efficacy of EGFR tyrosine kinase inhibitors or suppress tumor progression by blocking the lipidation and secretion of Hedgehog or Wnt ligands 6, 11. Consistent with this rationale, disruption of EGFR S-palmitoylation attenuates PI3K-AKT signaling and delays KRAS-driven lung tumorigenesis 12. These observations support palmitoylation-directed therapy as a mechanistically complementary approach that may be integrated with existing targeted and immune-based treatments.

Recent studies have further established that protein S-palmitoylation exerts multifaceted regulatory roles during tumor initiation and progression, participating broadly in signal transduction, metabolic reprogramming, and immune modulation 13. By controlling the localization, stability, and activation of membrane-associated proteins, this modification promotes tumor-cell survival, immune evasion, and metabolic adaptation 14. Functional genomic screens have identified several ZDHHC family members as “essential genes” required for cancer cell viability. For instance, a genome-wide siRNA screen by Tian et al. revealed that ZDHHC5 is a critical dependency factor for the growth and survival of non-small cell lung cancer (NSCLC) cells 15. Conversely, certain ZDHHC enzymes may play tumor-suppressive roles. Yeste-Velasco et al. demonstrated that ZDHHC14 undergoes frequent copy number loss or transcriptional downregulation in prostate and testicular germ cell tumors, and that its overexpression triggers caspase-dependent apoptosis, suggesting a tumor-suppressive function 16, 17. However, this role is not uniform across malignancies, as ZDHHC14 has also been reported to be activated in acute biphenotypic leukemia and in subsets of acute myeloid leukemia, indicating that its biological function may be context-dependent rather than uniformly tumor suppressive 18. Similarly, loss of ZDHHC13 function in mouse models leads to chronic cutaneous inflammation and constitutive NF-κB activation, markedly increasing the incidence of skin tumors, underscoring its essential role in tissue homeostasis and tumor suppression 19, 20. Together, these findings reveal a “double-edged” functional nature of the ZDHHC family across tissues and tumor types—acting either as promoters of tumorigenesis through stabilization of oncogenic signaling, or as safeguards that preserve cellular integrity and suppress malignant transformation.

Given its extensive involvement in oncogenic signaling and microenvironmental regulation, the protein palmitoylation machinery has recently emerged as a therapeutically actionable target. Multiple pharmacologic strategies have been developed to modulate this pathway. Early tool compounds—such as 2-bromopalmitate (2-BP) and the depalmitoylase inhibitor Palmostatin B—demonstrated the tractability of lipidation-dependent signaling, although their off-target activity limits mechanistic specificity 21, 22. In contrast, selective depalmitoylase inhibitors, including dimeric chloroquine (DC661) and Ezurpimtrostat (GNS561), specifically block PPT1, achieving precise suppression of depalmitoylation activity 23, 24. Competitive substrate-mimicking peptides have also been employed to interfere with enzyme-substrate interactions 25. Several newer approaches have shown mechanistically informative preclinical activity 23, 26; however, the current clinical experience remains limited 24, and for GNS561 the available evidence supports tractability of the PPT1 axis more clearly than objective efficacy or biomarker-defined benefit 24, 26. Targeting palmitoylation of immune-checkpoint proteins has further highlighted the immunologic relevance of this pathway. Yao et al. demonstrated that inhibiting PD-L1 S-palmitoylation promotes its ubiquitination and lysosomal degradation, reducing its membrane stability and thereby augmenting T-cell cytotoxicity 27. Moreover, the marine natural product Benzosceptrin C was found to selectively inhibit DHHC3 activity, similarly inducing PD-L1 degradation and enhancing anti-tumor immune responses in mouse models 28. Together, these studies support palmitoylation-directed intervention as a mechanistically relevant therapeutic strategy, while the detailed pharmacologic landscape is discussed in later sections.

Protein S-palmitoylation is now recognized as a pivotal regulatory axis in cancer progression, linking malignant signaling, metabolic remodeling, and immune evasion; however, its therapeutic relevance should be interpreted as a set of context-dependent, evidence-calibrated translational hypotheses rather than as a uniformly target-validating drug-development pathway 4, 29. Recent studies have further clarified the mechanistic continuum linking lipidation, signaling, and immunity, supporting a multi-tiered intervention framework centered on the ZDHHC family, substrate-specific modification sites, and the depalmitoylation axis 30, 31. Despite these advances, clinical translation remains constrained by catalytic conservation among ZDHHC enzymes, isozyme redundancy, and overlapping substrate repertoires 1, 4. Recent progress in structural and chemical biology, together with high-throughput screening platforms, has nevertheless created more practical entry points for selective inhibitor discovery and pharmacodynamic evaluation 32-34; however, these advances do not eliminate the central pharmacologic barriers posed by the conserved DHHC catalytic architecture, the membrane-embedded acyl-binding cavity, overlapping substrate repertoires, and the limited availability of substrate-level pharmacodynamic read-outs in clinically relevant specimens 29, 32, 33, 35-37. On the translational front, the depalmitoylation axis has already entered clinical evaluation. The PPT1 inhibitor GNS561 (Ezurpimtrostat) has shown acceptable early-phase tolerability and preliminary signals of disease stabilization in hepatocellular carcinoma 24; however, the available clinical experience should be interpreted as evidence of pharmacologic exposure and tolerability rather than proof of antitumor efficacy, and it does not yet establish objective response, validated predictive biomarkers, substrate-level target engagement, or a robust pharmacodynamic framework for patient selection 24. Mechanistic and tumor-microenvironmental studies support continued combination-based evaluation, but they do not substitute for prospective biomarker-linked clinical validation 26, 38. Against this background, the present review delineates the mechanistic landscape of ZDHHC-mediated S-palmitoylation in cancer, its cross-pathway coupling, and its therapeutically relevant nodes 1, 4, 29. Rather than treating the literature as a sequential catalogue of ZDHHC-substrate pairs, we organize the evidence around three analytical questions grounded in current methodological advances in substrate recruitment, in vitro reconstitution, and ZDHHC-specific S-acylation mapping 29, 35, 39, 40: which mechanisms are supported by convergent site-, enzyme-, pathway-, and phenotype-level validation; which associations remain provisional because of model restriction, incomplete rescue, or methodological ambiguity; and which unresolved controversies limit cross-tumor generalization or near-term therapeutic translation. We further discuss structure-guided small molecules, substrate-competitive peptides, depalmitoylase inhibitors, and their integration with immune-checkpoint blockade, metabolic intervention, and targeted therapy.

## 2. Structural Basis and Catalytic Mechanism of the ZDHHC Family: The Molecular Hub of Protein Palmitoylation

**Figure 1:**
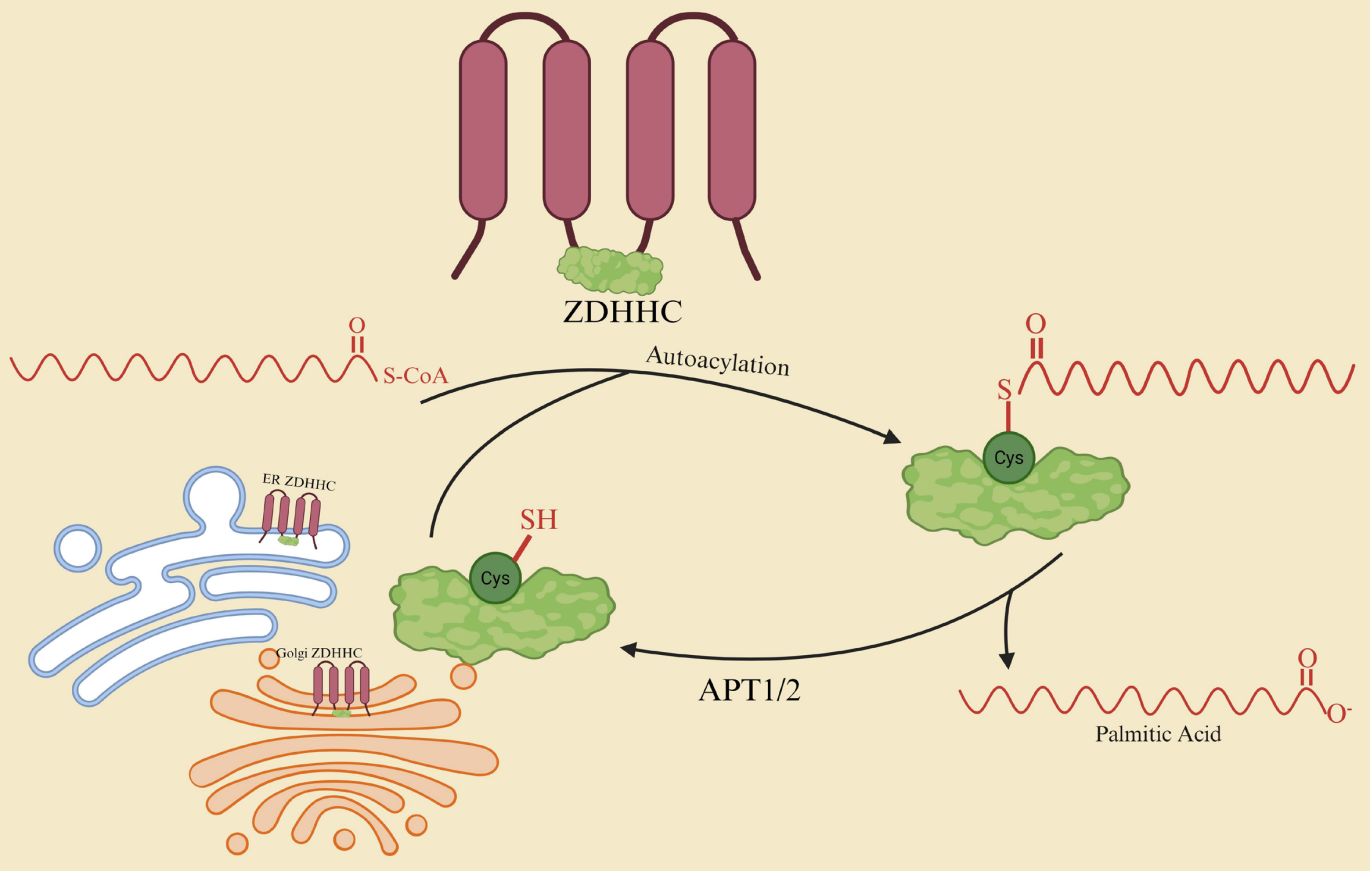
** Catalytic cycle of ZDHHC palmitoyltransferases and counter-regulation by APT1/2.** ZDHHC enzymes are multi-pass transmembrane proteins that localise predominantly to the endoplasmic reticulum and Golgi apparatus and use palmitoyl-CoA as an acyl donor. In the first “autoacylation” step, the conserved DHHC active-site cysteine forms a thioester acyl-enzyme intermediate. In the subsequent “transacylation” step, the acyl group is transferred to a cysteine residue on a cytosolic substrate, generating an S-palmitoylated protein that associates with cellular membranes. Acyl-protein thioesterases APT1 and APT2 then hydrolyse the thioester bond, releasing free palmitate and regenerating the unmodified cysteine, thereby completing the reversible palmitoylation-depalmitoylation cycle and dynamically tuning substrate membrane residency and signaling output.

Because the structural enzymology of ZDHHC proteins has been comprehensively reviewed elsewhere 29, 36, 37, 39, this section focuses only on the mechanistic features most directly relevant to cancer biology and therapeutic design. ZDHHC enzymes are multi-pass membrane acyltransferases whose catalytic core is defined by the Asp-His-His-Cys (DHHC) motif. They operate through a canonical two-step “autoacylation-transacylation” (ping-pong) mechanism at the cytosolic-membrane interface: first forming a thioester acyl-enzyme intermediate with fatty acyl-CoA, and then transferring the acyl group to a cysteine residue on the substrate protein to complete S-palmitoylation 41, 42. A schematic overview of this two-step autoacylation-transacylation cycle, together with the counter-regulatory depalmitoylation carried out by APT1/2, is illustrated in **Figure 1**. High-resolution structural analyses have revealed that human ZDHHC20 adopts a distinctive four-transmembrane, “tent-like” fold in which the helices form an enclosed hydrophobic cavity that accommodates the acyl chain and supports bivalent recognition of fatty acyl-CoA. This structural organization helps explain acyl selectivity, substrate engagement, and the continuing difficulty of achieving isoform-selective inhibition 32, 33, 36. Substrate selectivity is shaped by the combined effects of cavity geometry, motif-dependent substrate recruitment, and partner-guided localization. The hydrophobic cavity regulates acyl-chain access and transfer efficiency 32, 33, 36, whereas structural and motif-mapping studies of the DHHC17 ankyrin-repeat domain provide direct evidence for substrate engagement and motif-guided recruitment 43-45. In parallel, accessory proteins such as GOLGA7/GCP16 and SELENOK stabilize DHHC complexes and restrict their localization to the Golgi, ER, or plasma membrane, thereby enhancing catalytic efficiency and spatial fidelity 37, 46-48. At the regulatory level, ZDHHC activity is shaped by enzyme-enzyme cascades and enzyme-deacylase cross-talk. For example, ZDHHC16-mediated acylation of ZDHHC6 modulates the latter's stability and degradation sensitivity, producing a dynamic pool of interconvertible modification states 37. Conversely, depalmitoylases impose kinetic checkpoints: APT2 deforms membranes through its hydrophobic pocket to extract substrate acyl chains—defining the rate-limiting step of membrane de-anchoring 49—while the ABHD17 family specifically depalmitoylates N-Ras, tuning its membrane residency and signaling amplitude to maintain oncogenic threshold control 50. Technological advances in chemical biology and high-content imaging have completed the experimental pipeline from hit identification to mechanistic and cellular validation. Tools such as acyl-cLIP, in vitro reconstitution assays, and chemical-genetic tagging systems now allow high-resolution mapping of ZDHHC-substrate pairs 35, 40, and TR-FRET-based autoacylation readouts have accelerated the development of ZDHHC2 inhibitors 51. Integration of these structural, mechanistic, and methodological insights has empirically established the causal chain “site or acyl specificity → conformational control → membrane localization → signaling output” in cancer contexts. For instance, palmitoylation of the EGFR C-terminal tail fine-tunes PI3K-AKT signaling kinetics and receptor signal amplitude 11, 12, while S-palmitoylation of PD-L1 directly determines its membrane stability and immunosuppressive strength—and inhibition of this modification enhances T-cell cytotoxicity 27, 52.

Notwithstanding these advances, interpretation of the literature still requires substantial caution. A central methodological limitation is that many mechanistic claims are inferred from perturbations that do not isolate a single enzyme, substrate, cysteine site, cellular compartment, or modification stoichiometry. A meaningful fraction of the field still rests on non-selective pharmacology 21, 22, 53, 54, incomplete enzyme-substrate assignment 29, 35, 39, 40, limited endogenous-level validation, uncertain fractional occupancy of S-palmitoylation in vivo, and experimental settings in which trafficking state, protein abundance, forced overexpression, and subcellular compartment materially influence the apparent site and timing of S-palmitoylation 55-58. First, broad-spectrum tools such as 2-bromopalmitate and Palmostatin B lack sufficient specificity to assign enzyme-level causality on their own, because 2-BP perturbs both DHHC and depalmitoylase activities 21, 53, whereas Palmostatin B primarily targets APT1/2 and perturbs Ras localization while also producing broader proteomic effects 22, 54. Consequently, phenotypic rescue or suppression after treatment with these compounds should be interpreted as evidence of lipidation-cycle perturbation rather than as proof that a specific ZDHHC enzyme directly modifies a defined substrate site, unless paired with orthogonal genetic perturbation, catalytic rescue, and site-resolved substrate validation. Second, enzyme-substrate assignment remains incomplete in many studies that rely on overexpression, single-site mutagenesis, or bulk palmitoyl-proteomic enrichment without orthogonal rescue, a limitation emphasized by current work on substrate recruitment, in vitro reconstitution, and chemical-genetic mapping of ZDHHC-specific S-acylation 29, 35, 39, 40. Overexpression can increase substrate availability, alter trafficking routes, and force enzyme-substrate proximity, thereby creating apparent palmitoylation events that may not reflect endogenous catalytic relationships. Similarly, loss of signal after cysteine mutation or enzyme knockdown does not by itself establish direct enzyme-site causality, because the observed effect may arise from altered protein stability, localization, or pathway state. Third, modification stoichiometry remains difficult to quantify in vivo 29. Most commonly used enrichment-based assays, including acyl-biotin exchange, acyl-resin-assisted capture, click-chemistry profiling, and bulk palmitoyl-proteomics, are powerful for identifying candidate lipidated proteins but usually report relative enrichment rather than absolute fractional occupancy at endogenous sites 29, 35. Therefore, an apparent increase in palmitoylation may reflect altered protein abundance, labeling efficiency, compartmental enrichment, or sample processing rather than a true increase in in vivo modification stoichiometry 29, 35, 40. This limitation is especially relevant when palmitoylation is inferred from whole-cell lysates rather than compartment-resolved or endogenous-level assays 35, 55, 57, 58. Fourth, fundamental mechanistic questions remain unsettled, including the spatiotemporal assignment of palmitoylation across ER-Golgi-plasma membrane trafficking routes 55-58 and the extent to which autoacylation kinetics measured in cell-free systems reflect physiological catalytic control rather than assay-dependent intermediate accumulation 32, 33, 36, 51. Because ZDHHC enzymes, substrates, and depalmitoylases are compartmentally organized, cell-free activity, whole-cell palmitoyl-proteomic enrichment, and overexpression-based co-localization should not be assumed to represent the same catalytic event. Robust mechanistic inference requires convergence across endogenous localization, defined enzyme perturbation, site-resolved lipidation, and functional rescue in the same biological context. We therefore regard mechanistic conclusions as most secure when five layers of evidence converge within the same biological system: site-resolved detection or mutation, defined enzyme perturbation with catalytic rescue, endogenous-level substrate validation, compartment- or time-resolved localization, and restoration of the relevant pathway or phenotype by palmitoylation-competent but not palmitoylation-defective constructs.

## 3. Expression Profiles and Functional Rewiring of ZDHHCs in Cancer: Architects and Modulators of Oncogenic Networks

**Figure 2:**
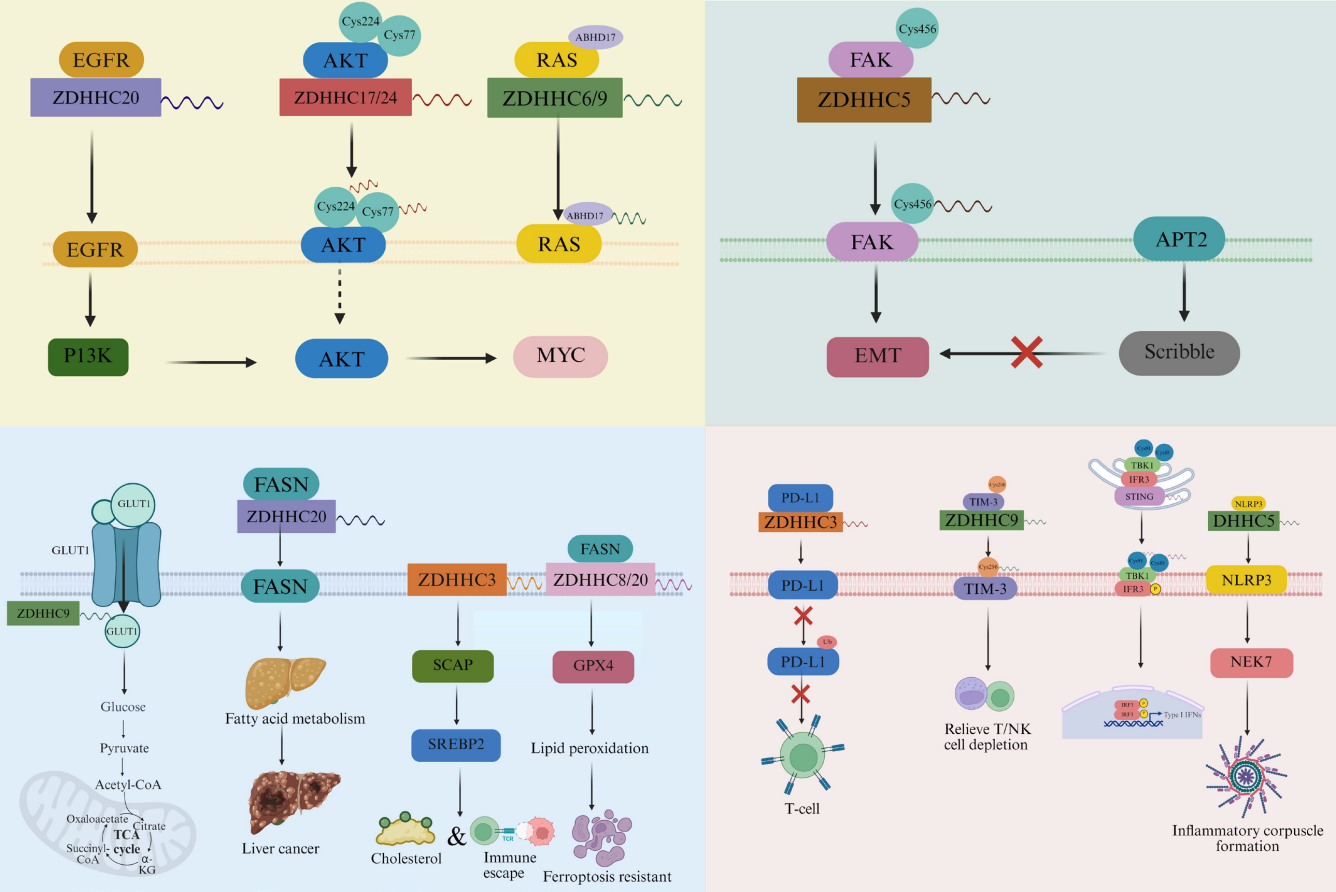
** ZDHHC-substrate signaling circuits integrating oncogenic pathways, metabolism and immunity.** Schematic overview of representative palmitoylation-depalmitoylation modules that couple ZDHHC activity to cancer-relevant signaling axes. **Top left:** ZDHHC20-mediated palmitoylation of the EGFR C-terminal tail and ZDHHC17/24-dependent modification of AKT at Cys77/Cys224 stabilise plasma-membrane anchoring and sustain PI3K-AKT-MYC signaling, whereas ZDHHC6/9-driven RAS palmitoylation is dynamically counter-balanced by the depalmitoylase ABHD17 to tune RAS output. **Top right:** ZDHHC5-catalysed palmitoylation of FAK at Cys456 promotes its membrane localization and kinase activation, driving epithelial-mesenchymal transition (EMT); in parallel, APT2-mediated depalmitoylation of the polarity protein Scribble facilitates its membrane release and further favours EMT. **Bottom left:** Metabolic rewiring nodes. ZDHHC9-dependent GLUT1 palmitoylation stabilises the transporter at the plasma membrane and enhances glycolytic flux; ZDHHC20-mediated FASN palmitoylation prevents its ubiquitin-proteasome degradation and supports lipogenesis; ZDHHC3-catalysed SCAP S-acylation activates SREBP2-driven cholesterol biosynthesis and immune evasion; and ZDHHC8/20-dependent GPX4 palmitoylation increases GPX4 stability, elevates the ferroptosis threshold and confers therapy resistance. **Bottom right:** Immune-checkpoint and innate-immunity modules. ZDHHC3-mediated palmitoylation of PD-L1 and ZDHHC9-mediated palmitoylation of TIM-3 stabilise these checkpoints at the plasma membrane, promoting T-cell and NK-cell exhaustion, whereas palmitoylation of STING and NLRP3 by DHHC enzymes licenses TBK1/IRF3 activation and NEK7-dependent inflammasome assembly, respectively, thereby shaping type I interferon signaling and inflammatory responses.

Across tumor types, multi-omic analyses consistently reveal that the ZDHHC-mediated S-palmitoylation network displays remarkable expression heterogeneity and subtype-specific remodeling. Its dysregulation aligns with key malignant programs—immune suppression, metabolic reprogramming, and stemness maintenance—thereby shaping both the strength and direction of tumor aggressiveness 59, 60. To anchor these observations at the level of individual enzymes, we summarize the subcellular localization, representative substrates, dominant signaling axes, and tumor phenotypes of the best-characterized ZDHHC members in a concise reference table **(Table 1)**. As summarized in an integrated ZDHHC-substrate signaling map **(Figure 2)**, these cross-pathway palmitoylation circuits converge on a limited set of oncogenic, metabolic, and immune nodes, including EGFR-AKT-RAS, FAK-EMT, GLUT1/FASN/GPX4, and PD-L1/TIM-3/STING/NLRP3. In lung adenocarcinoma (LUAD), integrative analyses of TCGA and multi-cohort datasets demonstrate that elevated ZDHHC4/12/18/24 and APT2 expression correlates with reduced CD8⁺ T-cell infiltration, increased CD276 (B7-H3) levels, and poor overall survival. These findings highlight the “palmitoylation-depalmitoylation axis” as a key regulator of the immune microenvironment and a potential stratification marker for patient prognosis 59. In glioblastoma (GBM), distinct members of the family show non-redundant specialization: ZDHHC18 and ZDHHC23 are enriched in complementary glioma stem-cell (GSC) niches, where they modulate BMI1-linked ubiquitination modules to establish “mesenchymal-like” and “neural-like” stemness programs 61. ZDHHC4, by palmitoylating GSK3β at Cys14, anchors the kinase at the plasma membrane, sustaining its activation and promoting temozolomide resistance and invasion 62. In contrast, ZDHHC16-mediated SETD2 palmitoylation imbalance disrupts H3K36me3 deposition and DNA-repair capacity, contributing to radioresistance 63. Furthermore, ZDHHC5 directly stabilizes EZH2 through site-specific palmitoylation, reinforcing the transcriptional programs that drive stemness and migration 64, while ZDHHC15-dependent GP130 lipidation enhances IL-6/STAT3 signaling—an effect that can be pharmacologically suppressed by local anesthetics to curb GSC self-renewal 65. Functionally, ZDHHC5 represents a bona fide oncogenic driver. Genome-wide loss-of-function screens and in vivo validations identify it as a growth-essential acyltransferase in non-small-cell lung cancer (NSCLC), whose inhibition impairs proliferation, migration, and xenograft growth 15. The ZDHHC5-FAK axis further links adhesion and migration signaling to palmitoylation dynamics: FAK S-palmitoylation determines its membrane localization and kinase activity, directly driving GBM invasion 66. At the receptor-lipid-raft interface, two complementary upstream paradigms have emerged. DHHC20-EGFR coupling exemplifies direct enzymatic control of receptor lipidation, whereas FASN-driven EGFR palmitoylation reflects a substrate-supply mechanism. Inhibiting DHHC20-mediated EGFR palmitoylation induces “TKI addiction,” sensitizing tumors to EGFR blockade, while FASN-dependent EGFR lipidation sustains metabolic dependence in EGFR-mutant NSCLC 11, 67. Notably, ZDHHC20-driven FASN palmitoylation prevents ubiquitin-mediated degradation of FASN, forming a positive feedback loop among lipogenesis, palmitoylation, and receptor stability 68. At the immune-checkpoint level, palmitoylation also acts as a decisive molecular switch. In this atlas-level section, PD-L1 is retained only as a representative high-evidence ZDHHC3-substrate circuit rather than re-discussed as a full immune-checkpoint mechanism. Among immune-checkpoint substrates, PD-L1 remains a representative and well-validated example of palmitoylation-dependent checkpoint stabilization 27, with DHHC3-targeted pharmacologic destabilization serving as a therapeutically informative extension of this paradigm 28. Detailed immunologic mechanisms and therapeutic implications are addressed in Sections 4.4 and 6, respectively. Multiple cross-axis circuits integrating metabolism, epigenetics, and immunity have now been experimentally validated, underscoring the translational potential of ZDHHC-mediated regulation. In colorectal cancer (CRC), ZDHHC6 palmitoylates PPARγ, promoting its nuclear translocation and activation of the ACLY-driven lipogenic axis, thereby elevating lipid flux and accelerating tumor growth 69. In hepatocellular carcinoma (HCC), ZDHHC23 palmitoylates and destabilizes PHF2, releasing the suppression of SREBP1c, reprogramming lipid metabolism, and promoting tumor progression 70. In pancreatic ductal adenocarcinoma (PDAC), ZDHHC9 palmitoylates LDHA, amplifying glycolysis and lactate production; this metabolic rewiring enhances responsiveness to PD-L1 blockade, linking energy metabolism to immune phenotype plasticity 71, 72. Crucially, in HCC, ZDHHC3-mediated SCAP palmitoylation disrupts SCAP/SREBP2 feedback inhibition, upregulating cholesterol biosynthesis and promoting immune evasion; combined targeting of ZDHHC3 and PD-1 achieves synergistic tumor suppression in vivo 73. Conversely, several ZDHHC members exert context-dependent tumor-suppressive or homeostatic roles. In prostate cancer, ZDHHC7 suppresses the androgen-receptor transcriptional program, and its loss correlates with aggressive progression 74. In murine models, Zdhhc13 deficiency compromises the epidermal barrier and triggers NF-κB-driven inflammation, predisposing to chemical carcinogenesis and highlighting its anti-inflammatory, tissue-protective function 75. Clinically, ZDHHC2 downregulation in gastric adenocarcinoma independently associates with lymph-node metastasis and poor prognosis 76, whereas in renal carcinoma, the same enzyme acts oncogenically by palmitoylating AGK, activating AKT-mTOR signaling, and reducing sunitinib sensitivity 77. In gastric cancer, ZDHHC2 also stabilizes NRF2 through palmitoylation, reinforcing antioxidant capacity and proliferative signaling 78. In clear-cell renal carcinoma (KIRC), ZDHHC3-mediated SLC9A2 palmitoylation regulates apoptosis 79, and pan-cancer analyses implicate ZDHHC18 in lipid metabolism and cuproptosis, suggesting prognostic relevance 80.

Collectively, these findings outline an atlas-level architecture rather than a second pathway-level discussion, comprising oncogenic driver axes and context-dependent suppressive/homeostatic axes with important implications for molecular stratification and combination therapy. At the driver level, ZDHHC3-SCAP, ZDHHC20-EGFR/FASN, ZDHHC9-LDHA/GLUT1, ZDHHC23-PHF2, and ZDHHC5-FAK/EZH2 serve as cross-axis anchors linking immune-metabolic, growth-factor, metabolic, epigenetic, invasion, and stemness programs 11, 12, 64, 66, 68, 70, 71, 73, 81. In contrast, ZDHHC7-, ZDHHC13-, and selected ZDHHC2/ZDHHC3-linked examples illustrate homeostatic or tumor-suppressive contexts, including androgen-receptor repression, epithelial-barrier maintenance/inflammation control, gastric-cancer clinical suppression, and SLC9A2-linked apoptosis 74-76, 79. Detailed pathway mechanisms are developed in Section 4, depalmitoylation dynamics in Section 5, therapeutic maturity in Section 6, and biomarker-guided stratification in Section 7.

Importantly, the evidentiary strength of ZDHHC-substrate circuits is not uniform across the field. We therefore propose a practical three-tier evidence framework for interpreting the current literature, drawing on recent advances in substrate recruitment analysis, in vitro reconstitution, and ZDHHC-specific S-acylation mapping 29, 35, 39, 40. Tier 1 mechanisms are supported by convergent site-level, enzyme-level, biochemical, functional, and in vivo evidence; representative examples include DHHC20-mediated EGFR palmitoylation 11, 12, PD-L1 stabilization through palmitoylation 27, 82, ZDHHC5-dependent FAK membrane localization and EMT signaling 66, ZDHHC3-mediated SCAP S-acylation in hepatocellular carcinoma 73, and the ZDHHC8/20-GPX4 ferroptosis-regulatory axis 83, 84. Tier 2 mechanisms have plausible enzyme-substrate or pathway-level support but remain dependent on a restricted set of models, incomplete rescue experiments, or limited endogenous validation 29, 35, 39, 40. Tier 3 associations are primarily inferred from expression correlations or pan-cancer signatures 85, single-model observations, or broad pharmacological perturbation using non-selective lipidation/depalmitoylation tools such as 2-bromopalmitate or APT inhibitors 21, 53, 54, 86, and should therefore be considered hypothesis-generating rather than target-validating. This tiered interpretation is particularly important for ZDHHC biology because the same enzyme may exert opposing functions across tumor types, as illustrated by context-dependent reports on ZDHHC14 and ZDHHC2 16-18, 76, 77; multiple ZDHHC isoforms may converge on overlapping substrate spaces, making enzyme-substrate assignment non-trivial 29, 35, 39; and altered palmitoylation may reflect changes in trafficking dynamics 55-58, substrate abundance or metabolic palmitate supply 67, 68, or broader cellular state rather than direct catalytic control. Where reproducibility across orthogonal model systems is lacking, such associations should be viewed as provisional rather than definitive. This distinction is critical when judging causality, cross-tumor generalizability, biomarker readiness, and near-term translational tractability.

## 4. Mechanistic Loops by Which ZDHHC-Mediated Palmitoylation Remodels Oncogenic Signaling and Cancer Stemness

### 4.1 Rewiring of Tumor Signaling Pathways and Maintenance of Cancer Stemness

ZDHHC-mediated S-palmitoylation converts site-specific lipid modification into sustained oncogenic signaling through three coordinated mechanisms: membrane anchoring, complex assembly, and stability control. ZDHHC9 stabilizes GLUT1 at the plasma membrane to sustain glycolytic flux 81, ZDHHC17 recruits the MAP2K4-JNK/p38 module to build activation scaffolds that amplify stress and stemness programs 87, and ZDHHC17-mediated Oct4A palmitoylation prevents lysosomal degradation and preserves its transcriptional activity 88. Representative substrate-pathway circuits that illustrate these layers and their actionable intervention points are summarized in **Table 2**. At the transcriptional and epigenetic level, Oct4A palmitoylation not only enhances protein stability but also cooperates with SOX4 to activate SOX2 enhancers, thereby sustaining the self-renewal and tumor-initiating capacity of glioblastoma stem cells (GSCs) 88. In p53-mutant gliomas, ZDHHC5 lipidates EZH2, forming an oncogenic EZH2-ZDHHC5 axis that drives GSC renewal and malignant progression 64. Under temozolomide (TMZ) resistance, ZDHHC4-mediated GSK3β (Cys14) palmitoylation enhances the EZH2-STAT3 pathway, reinforcing stemness and chemoresistance—identifying ZDHHC4 as a functional “resistance-stemness coupling node” 62. In EGFR-altered GBM, suppression of the ZDHHC16-SETD2-H3K36me3 axis weakens DNA-damage repair following radiotherapy. Pharmacologic inhibition of depalmitoylation using PalmB partially restores this pathway, revealing a drug-responsive “palmitoylation-chromatin-repair” interface 63.

At the receptor-cytokine level, the CK2α-ZDHHC15-c-MET cascade directly enhances c-MET glycosylation, dimerization, and activation through site-specific palmitoylation, forming a central oncogenic signaling hub 89. Conversely, clinically used local anesthetics suppress ZDHHC15 expression and block GP130 palmitoylation, thereby attenuating the IL-6/STAT3 cascade and limiting GSC self-renewal 65. Within the membrane-adhesion-cytoskeleton dimension, ZDHHC5-mediated FAK (C456) palmitoylation governs its membrane anchoring and kinase activation, coupling adhesion signaling to EMT and invasion programs 66. This “receptor or adhesion lipidation → sustained signal → stemness amplification” paradigm interlocks with downstream lipidation-dependent transcriptional feedback circuits—for example, STAT3 palmitoylation, which amplifies oncogenic transcriptional output 90. This lipidation logic is also validated in receptor-kinase systems beyond glioma. EGFR palmitoylation ensures efficient plasma-membrane targeting and proper signaling amplitude 6, while AKT palmitoylation at C77/C224, catalyzed by ZDHHC17/24, allows PIP3-independent anchoring and persistent activation, augmenting oncogenic signaling and promoting tumorigenesis 91.

Within the highly drug-relevant RAS/MAPK signaling axis, a clear chemical intervention paradigm has emerged for directly disrupting PAT-RAS coupling. The antimalarial compound artemisinin covalently binds and inhibits the ER-resident palmitoyltransferase ZDHHC6, thereby reducing NRAS S-palmitoylation, mislocalizing NRAS from the plasma membrane, and concurrently suppressing ERK and AKT signaling cascades 92. A parallel mechanism couples vesicular transport and palmitoylation through the small GTPase RAB27B, which recruits ZDHHC9 to promote NRAS palmitoylation and membrane anchoring—maintaining high c-RAF/MEK/ERK activity. In AML patient cohorts and murine models, RAB27B inhibition downregulates ERK signaling and suppresses leukemic progression 93. The opposing depalmitoylation valve of this circuit is equally druggable. The selective ABHD17 inhibitor ABD957 blocks NRAS depalmitoylation and recycling, reducing its plasma-membrane residency and attenuating downstream signaling; importantly, ABD957 shows synergistic antitumor activity when combined with MEK inhibitors 94. At the receptor level, palmitoylation of the estrogen receptor β (ERβ) promotes its interaction with caveolin-1/p38-MAPK, triggering a pro-apoptotic signaling loop—demonstrating that palmitoylation-MAPK coupling can also establish tumor-suppressive circuits and providing mechanistic leverage for pathway-specific therapeutic design 95.

From a protein-protein interaction perspective, ZDHHC17 uses its ankyrin repeat (ANK) domain to tether MAP2K4, assembling a functional JNK/p38 signaling complex that sustains GSC self-renewal. The small molecule genistein specifically disrupts this interaction, inhibits glioblastoma growth, and validates non-catalytic enzyme-substrate interfaces as novel druggable sites 87. Finally, in RAS-driven leukemogenesis, the Cys180 palmitoylation and KIKK membrane-targeting motif of KRAS4A act cooperatively to define its transforming potential. Dual disruption of these motifs nearly abolishes tumorigenicity, providing compelling experimental proof that the PAT-RAS axis represents a mechanistically tractable and therapeutically actionable vulnerability 96.

The PI3K/AKT signaling cascade is finely tuned by site-specific palmitoylation, which dictates both lipid-raft localization and noncanonical activation dynamics. ZDHHC17/24-mediated S-palmitoylation of AKT at Cys77 and Cys224 enables stable plasma-membrane anchoring under PIP3-independent conditions, preventing the formation of inactive cytosolic aggregates and ensuring sustained kinase activation 91. Under high-fat dietary or lipotoxic stress, this palmitoylation switch accelerates the pathological progression from nonalcoholic steatohepatitis (NASH) to hepatocellular carcinoma (HCC), whereas genetic knockout or cell-penetrating peptide blockade of the modification markedly attenuates this transition 91. At the coupling interface of upstream and downstream modules, the ZDHHC2-AGK palmitoylation axis activates the AKT-mTOR pathway and reduces the sensitivity of clear-cell renal carcinoma (ccRCC) to sunitinib, highlighting its role in therapeutic resistance 77. In contrast, ZDHHC22-mediated palmitoylation of mTOR decreases its stability and dampens AKT/mTOR signaling kinetics, thereby inhibiting breast cancer proliferation and reversing endocrine therapy resistance 97. At the receptor level, ZDHHC20-catalyzed palmitoylation of the EGFR C-terminal tail promotes the ordered assembly of the PI3K complex, maintaining the PI3K-AKT-MYC signaling axis 12. In KRAS-mutant NSCLC, inhibition of this modification downregulates PI3K-AKT signaling, suppresses MYC expression, and markedly enhances sensitivity to PI3K inhibitors 12. Mechanistically, this effect involves conformational remodeling of the disordered EGFR C-terminal tail and its binding spectrum with p85 and Grb2, achieving a functional bifurcation between the PI3K and MAPK branches. This observation aligns with the structural model wherein palmitoylation constrains receptor-tail conformation and fine-tunes signaling amplitude 6. Collectively, the “site modification-membrane timing-signal amplitude” triad of AKT and EGFR establishes a regulatory framework that determines oncogenic signaling persistence and therapeutic responsiveness. This conceptual model supports the rational combination of PAT inhibitors or substrate-competitive peptides with PI3K inhibitors, endocrine therapy, or immunotherapy, and positions palmitoylation fingerprinting as a promising tool for precision stratification and response monitoring in targeted cancer therapy.

Site-specific palmitoylation directly defines both the cell-death threshold and the starting point of vesicular trafficking in programmed cell death and autophagy. In hepatocellular carcinoma (HCC), lenvatinib enhances GSDME S-palmitoylation and membrane localization, triggering pyroptosis and thereby increasing drug sensitivity 98. For the pyroptotic executor GSDMD, a recent multicenter study demonstrated that ROS-induced S-palmitoylation at Cys191 (human) / Cys192 (mouse)—catalyzed by ZDHHC5 and ZDHHC9—is an essential prerequisite for membrane translocation, oligomerization, and pore formation; inhibition of this modification reduces IL-1β release and improves survival in sepsis models 99. Proteomic and functional analyses further revealed that FASN forms a complex with GSDMD, promoting its lipid modification, while palmitoylation of GSDMD-NT directly determines its membrane affinity and pore-forming activity 100. Independent studies have confirmed that S-palmitoylation at the conserved Cys191 site is sufficient to amplify GSDMD-mediated pyroptosis and cytokine release, consolidating the causal chain of “site → localization → function” 101. In autophagy initiation, ZDHHC7-mediated palmitoylation of ATG16L1 at Cys153 enhances its interaction with WIPI2B/RAB33B, facilitating LC3 lipidation and autophagosome assembly 102. Concurrently, ZDHHC5-catalyzed S-palmitoylation of ATG9A at Cys155/156 drives its docking to the AP-4ε complex and coordinates directed trafficking from the Golgi to the nascent autophagic membrane, thereby initiating autophagy at its source 103. Within the apoptotic pathway, BAX undergoes site-dependent palmitoylation that enhances its mitochondrial membrane anchoring and pro-apoptotic activity, forming an intervention-ready cascade linking “site modification → membrane localization → death threshold” 104. In tumor-suppressive circuits, ZDHHC1-mediated S-palmitoylation of p53 (C135/C176/C275) is indispensable for its nuclear translocation and tumor-suppressive function. Moreover, p53-DNMT3A-dependent methylation of the ZDHHC1 promoter establishes a negative feedback loop that interlinks palmitoylation, epigenetic regulation, and DNA repair/apoptosis homeostasis 105.

At the metabolism-stemness interface, site-specific S-palmitoylation directly links membrane anchoring, complex assembly, and protein stability/turnover to metabolic adaptation and cellular plasticity. In glioblastoma (GBM), ZDHHC9-mediated palmitoylation of GLUT1 at Cys207 stabilizes its plasma-membrane localization, enhances glucose uptake and glycolytic flux, and thereby drives tumorigenesis and progression 81. In pancreatic ductal adenocarcinoma (PDAC), ZDHHC9-dependent palmitoylation of LDHA alters its enzymatic conformation and reshapes chemosensitivity profiles, highlighting a tractable “lipidation-metabolism-drug response” axis 71. In colorectal cancer (CRC), the ZDHHC6-PPARγ-ACLY cascade stabilizes and promotes nuclear translocation of PPARγ, upregulating fatty-acid synthesis and lipidomic reprogramming to support tumor growth 69. Similarly, in hepatocellular carcinoma (HCC), ZDHHC23 facilitates palmitoylation-dependent ubiquitination and degradation of PHF2, thereby releasing SREBP1c suppression and rewiring lipid metabolism 70. In parallel, ZDHHC20-mediated palmitoylation of FASN stabilizes the enzyme by preventing ubiquitin-proteasome degradation, reinforcing a “palmitate supply-lipogenesis-membrane biogenesis” feedback loop that fuels hepatocarcinogenesis 68. Collectively, these data outline a reproducible mechanistic chain—“metabolic substrate or enzyme lipidation → bioenergetic and membrane flux → stemness and adaptive capacity”—and show that, under systemic metabolic stress (e.g., high-fat diet), AKT palmitoylation further extends this network to drive the transition from NASH to HCC 91.

At the immune-stemness interface, palmitoylation governs the stability of membrane checkpoint proteins that serve as molecular gates for immune evasion. PD-L1 S-palmitoylation, mainly catalyzed by ZDHHC3, stabilizes its membrane residency and promotes immune escape; inhibition of this modification triggers ubiquitination-coupled lysosomal degradation and enhances T-cell cytotoxicity 27. The marine natural compound Benzosceptrin C directly targets DHHC3, inducing PD-L1 depalmitoylation and degradation, which markedly boosts antitumor immunity in vivo 28. Beyond PD-L1, TIM-3 palmitoylation, mediated by ZDHHC9, maintains the exhausted T/NK-cell phenotype, whereas its blockade relieves immunosuppression 106. In innate immunity, STING palmitoylation at the Golgi is essential for TBK1/IRF3 recruitment and type I interferon (IFN-I) activation 107, while ZDHHC5-mediated NLRP3 palmitoylation enhances NEK7 binding and inflammasome assembly 108. Conversely, corrective “site-level depalmitoylation” can potentiate antitumor immunity: inhibition of LYPLAL1-mediated cGAS depalmitoylation amplifies its activation and increases responsiveness to immune-checkpoint blockade 109. In the metabolism-immunity dimension, suppression of ZDHHC3-SCAP palmitoylation disrupts the cholesterol-synthesis feedback loop and synergizes with anti-PD-1 therapy to achieve enhanced tumor control 73. Together, these findings identify palmitoylation as a bidirectional molecular switch coordinating metabolic reprogramming, stemness, and immune evasion. Strategies based on PAT inhibition, substrate-competitive peptides, or depalmitoylase targeting therefore offer actionable intervention routes, forming mechanism-driven combinations that simultaneously modulate pathway kinetics and immune phenotypes for next-generation precision therapy.

### 4.2 The Palmitoylation Switch in Tumor Invasion and Metastasis

Protein S-palmitoylation acts as a multi-tiered molecular relay—membrane anchoring → complex assembly → signal coupling—that orchestrates key processes including cell adhesion, cytoskeletal remodeling, epithelial-mesenchymal transition (EMT), and microenvironmental colonization. Together, these mechanisms establish a causal sequence of “site modification → membrane/lipid-raft localization → signaling activation → invasion and metastasis” 4. Along the receptor/adhesion axis, functional palmitoylation of GRK6 has been identified in triple-negative breast cancer (TNBC), where it amplifies migration and EMT through β-Arrestin2/MAPK/NF-κB recruitment. Genetic silencing or site-specific blockade of this modification markedly suppresses metastatic potential, defining a therapeutically accessible node 110. The natural compound curcumin inhibits palmitoylation of integrin β4 (ITG-β4), disrupting its lipid-raft localization and downstream signaling, while concurrently inhibiting ZDHHC3 autoacylation, resulting in a dual “enzyme-substrate” blockade that significantly reduces tumor cell invasion and migration 111. In scirrhous-type gastric cancer, ZDHHC14 is upregulated and promotes invasion through a coupled adhesion-protease pathway: its inhibition reduces MMP-17 expression and activity and downregulates ITGA5/ITGB1, collectively impairing metastatic competence 112. Functional screens in NSCLC have identified ZDHHC5 as a “growth/migration-dependent” palmitoyltransferase whose catalytic activity is indispensable; inhibition of ZDHHC5 simultaneously attenuates proliferation, clonogenicity, and invasion 15. At the lipid-raft level, multi-site palmitoylation of KAI1/CD82, a metastasis suppressor, is essential for its membrane anchoring and anti-invasive function 113. Conversely, CD44 palmitoylation dictates its residency within lipid rafts, whereas depalmitoylation redirects it to non-raft domains, enhances its interaction with ezrin, and increases EMT and migratory potential 114-116. At the tight junction and adhesion complex level, the ZDHHC12-CLDN3 palmitoylation triad stabilizes CLDN3 at the plasma membrane, driving ovarian cancer progression and invasion 117. As a key adhesion-signaling hub, FAK palmitoylation—catalyzed by ZDHHC5—is required for its plasma-membrane localization and kinase activation, thereby linking local adhesion clusters to migratory and invasive programs 66. Likewise, palmitoylation of the membrane-scaffold protein Flotillin-1 is indispensable for its stability and pro-migratory functions; genetic or pharmacologic inhibition of this lipidation downregulates invasive signaling, confirming the general framework of a “raft-signal-migration” axis 118. Beyond adhesion receptors, small GTPases that control cytoskeletal dynamics also depend on lipidation switches. Rac1 activity is governed by a reversible “palmitoylation-depalmitoylation” cycle that regulates its membrane partitioning and cytoskeletal remodeling; loss of palmitoylation directly impairs migration 119. Similarly, in clinically relevant colorectal cancer models, inhibition of Rap2b S-palmitoylation relocalizes the protein from the plasma membrane to the cytosol, thereby suppressing metastatic dissemination 120. In summary, site-specific S-palmitoylation functions as an upstream molecular switch controlling tumor invasion and metastasis. Targeting this regulatory layer—through PAT inhibition, depalmitoylase modulation, or substrate-competitive peptides, and in rational combination with metabolic or immune-based therapies—offers a strategy for multi-site, reversible, and precise intervention across the adhesion-raft-signaling-migration/metastasis axis 4.

Within the cytoskeleton-polarity axis of EMT regulation, androgens markedly increase the S-palmitoylation of α-tubulin in prostate cancer-derived LNCaP cells, enhancing its plasma-membrane anchoring. Inhibition of this modification by 2-bromopalmitate (2-BP) suppresses cell proliferation, delineating a causal chain of “cytoskeleton lipidation → malignant phenotype” 121. It is worth emphasizing that 2-BP is not a selective DHHC inhibitor and also blocks the depalmitoylases APT1/2, a pharmacologic caveat that must be considered when interpreting such data 53. During EMT induction, Snail upregulates APT2 while concurrently downregulating several zDHHC enzymes, leading to Scribble depalmitoylation and membrane detachment. Selective inhibition of APT2 restores Scribble to the plasma membrane and suppresses MEK-MAPK activation, thereby functioning as a rate-limiting valve for polarity and migration signaling 122. The small GTPase Rap2b depends on C-terminal di-cysteine (C176/C177) S-palmitoylation for plasma-membrane residency; blocking this modification—by competitive peptides or chemical inhibitors—removes Rap2b from the membrane and restricts colorectal cancer cell migration and metastasis. This process is governed by the EGFR/PI3K-ABHD17a axis 120. At the adhesion-signaling hub, FAK palmitoylation at Cys456, catalyzed by ZDHHC5, maintains membrane anchoring and kinase activity. Either ZDHHC5 knockdown or a C456S mutation attenuates EMT marker expression and invasion, defining a self-reinforcing ZDHHC5-FAK regulatory loop 66. At the polarity-maintenance layer, ZDHHC7-mediated Scribble palmitoylation is indispensable for its membrane localization and polarity stabilization, implying that a “depalmitoylation → de-anchoring → polarity collapse” sequence can serve as an upstream EMT trigger 123. In the metabolism-cytoskeleton interface, endogenous palmitate supplied by FASN supports RhoU S-palmitoylation and stabilizes Cdc42, promoting adhesion turnover and migration; exogenous palmitate partially rescues this complex, directly linking “fatty-acid synthesis → lipidation → cytoskeletal remodeling → migration.” 124 Collectively, site-specific S-palmitoylation of critical substrates—α-tubulin, Rap2b, FAK, and Scribble—clarifies the closed mechanistic loop of “site → membrane/lipid-raft → pathway → invasion/metastasis.” These nodes constitute an actionable target cluster for PAT inhibition, depalmitoylase blockade, or substrate-competitive peptides, offering concrete leverage points for anti-metastatic intervention 7.

At the nutritional-mechanotransduction interface, dietary palmitic acid amplifies YAP signaling through a ZDHHC15-KIBRA-YAP positive-feedback loop. ZDHHC15 catalyzes KIBRA S-palmitoylation, promoting its degradation and thereby relieving suppression of YAP. This cascade markedly enhances metastatic potential in both breast and ovarian cancer models, defining an actionable “nutrition-lipidation-mechanotransduction” axis 125. At the microbiome level, Fusobacterium periodonticum engages FLOT1 via its adhesin FadA-like protein (FadAL), activating the PI3K-AKT/FASN pathway and driving intracellular palmitate accumulation. This, in turn, increases Wnt3A S-palmitoylation at Cys77 and promotes its membrane targeting, initiating Wnt/β-catenin-driven EMT subtypes that fuel invasion and metastasis in esophageal squamous cell carcinoma (ESCC) 126. At the immune-organotropism interface, in-vivo functional screening identifies ZDHHC20 as a key driver of distant metastasis in pancreatic ductal adenocarcinoma (PDAC). ZDHHC20 upregulation renders tumor cells resistant to NK-cell cytotoxicity and remodels their membrane proteome through substrate-specific lipidation, establishing a “palmitoylation-immune evasion-organ colonization” feedback loop 127. Conversely, ZDHHC11B is downregulated in lung adenocarcinoma and acts as a tumor suppressor by restraining EMT and reducing migration and invasion, underscoring the context-dependent bidirectionality of the ZDHHC family 128. More broadly, high-fat diets induce AKT S-palmitoylation at Cys77/Cys224, enabling PIP3-independent membrane anchoring and activation, which amplifies the oncogenic PI3K/AKT cascade during NASH-to-HCC progression—reinforcing the general paradigm of “nutrition-driven lipidation as a pro-oncogenic and pro-metastatic foundation.” 91 Consistent human and animal data further show that dietary palmitate, acting through CD36, selectively enhances the metastatic competency of metastasis-initiating cells, providing convergent mechanistic and clinical evidence for the concept of “nutrient input → metastatic susceptibility.” 129, 130 At the metabolism-lipidation coupling end, ZDHHC20-mediated FASN S-palmitoylation stabilizes the enzyme by preventing its ubiquitin-proteasome degradation, thereby driving hepatocarcinogenesis and establishing a “PAT-metabolic enzyme” axis that fuels malignant progression from the lipid-supply side 68.

### 4.3 Metabolic Reprogramming: Adaptive Metabolic Switches in Tumor Cells

Tumor metabolism operates through a multi-axis coupling of glycolysis, lipid synthesis/oxidation, mitochondrial respiration, and redox or ferroptotic control, with S-palmitoylation functioning as a site-specific “gear-shift” that translates nutrient flux into signaling output and adaptive phenotypes in real time 4, 85. At the level of glucose uptake, ZDHHC9-mediated palmitoylation of GLUT1 at Cys207 anchors the transporter at the plasma membrane, enhancing glucose import and glycolytic flux, thereby promoting glioblastoma initiation and progression 81. At the enzyme-activity switch, ZDHHC9-dependent palmitoylation of LDHA (Cys163) alters its catalytic conformation and tetramer stability, reshaping chemosensitivity in pancreatic ductal adenocarcinoma (PDAC) and illustrating a pharmacologically tractable “lipidation-metabolism-drug-response” link 71. In lipogenesis and lipid-droplet biogenesis, the ZDHHC6-PPARγ-ACLY cascade upregulates de novo lipid synthesis and remodels the lipidome to drive colorectal-cancer progression 69. Similarly, ZDHHC23-mediated palmitoylation of PHF2 triggers its ubiquitination and degradation, relieving SREBP1c suppression and globally reprogramming lipogenic transcription in hepatocellular carcinoma (HCC) 70. Within the positive feedback loop of “palmitate supply-lipogenesis-membrane biogenesis,” ZDHHC20 palmitoylates FASN (C1471/C1881), stabilizing the enzyme against ubiquitin-proteasome degradation and thereby promoting hepatocarcinogenesis 68. At the physiologic-metabolic interface, a high-fat diet induces AKT palmitoylation (Cys77/Cys224) and plasma-membrane anchoring, producing sustained PIP3-independent activation that drives the oncogenic transition from NASH to HCC 91. In EGFR-mutant NSCLC, de novo palmitate synthesized by FASN maintains EGFR palmitoylation and continuous signaling; inhibition of FASN destabilizes EGFR and suppresses the growth of drug-resistant cells, exemplifying a reciprocal “lipid metabolism-palmitoylation-growth advantage” circuit 67. In parallel, EGFR palmitoylation directly controlled by ZDHHC20 establishes a pharmacologic vulnerability: disrupting this modification induces EGFR-TKI dependency and defines a rational entry point for combination therapy at the PAT level 11. More broadly, S-palmitoylation of GPX4 modulates lipid-peroxidation dynamics and ferroptosis sensitivity, coupling redox balance with antitumor immunity and positioning palmitoylation as a central node linking glycolytic, lipid, and ferroptotic axes 83. Collectively, evidence from mechanistic and disease models consolidates the causal chain “substrate/enzyme lipidation → energy and membrane flux → growth advantage and therapeutic response.” This framework provides a solid mechanistic basis for combinatorial strategies integrating PAT inhibition, depalmitoylase modulation, or substrate-competitive peptides with metabolic intervention 4, 85.

Within the ferroptosis and oxidative-stress axis, S-palmitoylation serves as a sequential regulator of “site modification → membrane anchoring → protein stability,” establishing the antioxidant defense threshold and ferroptosis sensitivity that determine tumor-cell survival under therapeutic or immune stress 83, 84. In hepatocellular carcinoma (HCC), the long noncoding RNA DUXAP8 promotes S-palmitoylation of SLC7A11, preventing its lysosomal degradation and stabilizing the transporter at the plasma membrane. This process inhibits ferroptosis and induces resistance to sorafenib 131. Cross-cancer analyses further reveal that ZDHHC8, activated by AMPKα1 phosphorylation, enhances SLC7A11 palmitoylation and stability, thereby reinforcing resistance to ferroptotic stress—a broadly conserved “SLC7A11-palmitoylation” pro-survival axis 132. At the depalmitoylation checkpoint, the ABHD17 family acts as a key enzymatic system mediating the depalmitoylation cycle of plasma-membrane proteins, including N-Ras. Its selective inhibitor, ABD957, blocks N-Ras depalmitoylation and downstream oncogenic signaling, providing a chemical handle to manipulate oxidative-stress-related pathways through depalmitoylase inhibition 50, 94. From the antioxidant-enzyme perspective, inhibition of the depalmitoylase PPT1 (e.g., by DC661) enhances Gpx1 S-palmitoylation and protein stability, leading to potent suppression of pathological angiogenesis. This establishes a pharmacologically actionable “depalmitoylation-palmitoylation homeostasis-ROS/angiogenesis” axis 133. Most recently, GPX4 itself was demonstrated to undergo reversible S-palmitoylation: ZDHHC20-mediated lipidation at Cys66 stabilizes GPX4 and suppresses ferroptosis, whereas disruption of this modification lowers the ferroptotic threshold and sensitizes tumors to therapy 84. In parallel, ZDHHC8-catalyzed GPX4 palmitoylation (Cys75) dictates ferroptosis sensitivity and amplifies CD8⁺ T cell-driven lipid peroxidation, enhancing antitumor immune responses. Together, these findings highlight GPX4 palmitoylation as a pharmacologically accessible lever that coordinates oxidative defense, ferroptosis, and immune-mediated cytotoxicity 83.

In oral squamous cell carcinoma (OSCC), ferroptosis is increasingly recognized as a therapeutically relevant vulnerability, particularly in cisplatin-resistant and redox-adapted states 134-136. Although direct evidence linking individual ZDHHC enzymes to ferroptosis control in OSCC remains limited, palmitoylation-regulated signaling is already operative in this disease. Specifically, RAB27A promotes migration and invasion in OSCC by enhancing ZDHHC13-dependent EGFR palmitoylation and membrane stability 137, whereas ferroptosis sensitivity in OSCC can be shaped by ACSL3/GPX4-centered antioxidant control 135. These observations suggest that ZDHHC-dependent signaling and ferroptosis-defense pathways may intersect in OSCC and therefore warrant direct mechanistic investigation.

At the mitochondrial level, ZDHHC18 directly catalyzes the S-palmitoylation of mitochondrial malate dehydrogenase (MDH2) at Cys138, enhancing its enzymatic activity and sustaining oxidative phosphorylation. This modification accelerates ovarian cancer cell growth and progression, defining a mechanistic bridge between palmitoylation and mitochondrial respiration 138. Within the cholesterol-Wnt crosstalk axis, cholesterol specifically binds to the extracellular linker domain of Frizzled-5 (FZD5), triggering S-palmitoylation at Cys538, which promotes receptor maturation and trafficking. This lipidation sustains the dependency of RNF43-mutant pancreatic tumors on FZD5-mediated Wnt/β-catenin signaling, whereas the endogenous oxysterol 25-hydroxycholesterol (25-OHC) competitively blocks this binding and disrupts the “cholesterol-FZD5-palmitoylation” circuit 139. Genome-wide CRISPR screening has independently validated this FZD5-dependent vulnerability as a druggable liability in RNF43-mutant pancreatic cancer 140. In colorectal cancer, ACOX1 dephosphorylation and degradation lead to palmitate accumulation, which drives β-catenin S-palmitoylation at Cys466, inhibits its ubiquitin-mediated degradation, and amplifies Wnt signaling—forming a metabolic feedback loop of “lipid metabolism → palmitoylation → signaling reinforcement.” 141 From a structural perspective, crystallographic analyses have revealed that the Wnt lipid moiety directly fits into the hydrophobic groove of Frizzled's cysteine-rich domain (CRD), providing atomic-level evidence for the “lipidation-receptor-signaling” causal chain 142. Complementary biochemical work further demonstrates that Wnt biogenesis and secretion are tightly controlled by PORCN-mediated acylation and coordinated assembly with the chaperones Wntless (WLS) and calreticulin (CALR), which together govern the assembly, trafficking, and maturation of lipidated Wnt ligands 143. These findings firmly anchor metabolic lipid flux as a structural and functional prerequisite for Wnt-driven oncogenic signaling.

Recent clinical and mechanistic evidence identifies ZDHHC7 as a pivotal lipidation regulator in ovarian clear cell carcinoma (OCCC). By suppressing YAP1 activity, ZDHHC7 confers resistance to lipid peroxidation and ferroptosis, correlating strongly with poor prognosis. These findings define a functional Hippo-ferroptosis lipidation nexus with direct clinical relevance 144. Complementing this, auto-palmitoylation of TEAD transcription factors is essential for YAP/TAZ binding and transcriptional activation. Structural studies reveal that the central hydrophobic pocket of TEAD accommodates a palmitoyl chain, stabilizing the protein fold and maintaining its transcriptional competence 145, 146. The palmitoylation state of TEAD is dynamically modulated by cell-cell contact and the Nf2/Merlin-fatty-acid-synthesis/depalmitoylase axis (APT2, ABHD17), directly linking cellular metabolic status to Hippo transcriptional output 147. Leveraging this “lipid pocket,” a growing array of small molecules now target TEAD lipidation to inhibit oncogenic YAP/TAZ-TEAD signaling. These compounds—ranging from autopalmitoylation blockers to allosteric pocket occupiers—effectively disrupt YAP/TAZ-TEAD interactions. Notably, inhibitors such as GNE-7883 and IAG933 have shown robust antitumor efficacy in NF2-deficient and therapy-resistant tumor models, providing compelling structural and pharmacologic validation for the “metabolism-lipidation-Hippo” axis 148-150. From a functional proteomic and metabolomic standpoint, ZDHHC2 has been identified as the key palmitoyl acyltransferase (PAT) responsible for the lipidation of substrates such as CKAP4, maintaining its plasma-membrane localization and antiproliferative signaling, thereby suggesting a context-dependent tumor-suppressive role 151, 152. Complementary metabolomic analyses demonstrate that manipulating DHHC2 expression reshapes the cellular metabolite landscape: overexpression increases levels of anticancer metabolites such as betaine and 5′-methylthioadenosine, whereas knockdown enriches pro-oncogenic metabolic intermediates. These data reinforce a causal framework linking lipidation, metabolic remodeling, and tumor phenotype 153. On the depalmitoylation axis, the ABHD17 family serves as plasma-membrane depalmitoylases that selectively remodel the dynamic palmitoyl-proteome and attenuate NRAS-driven signaling and tumor growth, forming a parallel regulatory equilibrium with ZDHHC2. This lipidation-depalmitoylation balance highlights convergent therapeutic entry points for combined or sequential interventions targeting both arms of the palmitoylation cycle 94.

### 4.4 Membrane Protein Stabilization and Signaling Remodeling in Immune Evasion

At the immune-checkpoint level, S-palmitoylation acts as a “membrane stability switch” that directly determines the surface retention and inhibitory potency of immune receptors and ligands 27, 82. PD-L1 palmitoylation, primarily catalyzed by ZDHHC3, prevents its ubiquitination and lysosomal degradation, thereby enhancing plasma-membrane stability and immune evasion. Blocking this modification restores PD-L1 turnover and significantly boosts T-cell cytotoxicity 27, 82. The marine natural product Benzosceptrin C selectively inhibits DHHC3, inducing lysosomal degradation of PD-L1 and strengthening antitumor immunity in vivo—validating a “enzyme inhibition → depalmitoylation → destabilization” pharmacologic axis 28. On the receptor side, TIM-3 undergoes ZDHHC9-mediated palmitoylation at Cys296, which protects it from HRD1-dependent ubiquitination, stabilizes its membrane presence, and sustains immune exhaustion. A site-specific blocking peptide accelerates TIM-3 degradation and enhances CAR-T and NK-cell function 106. Similarly, a designed PD-1 blocking peptide (PD1-PALM) competitively inhibits PD-1 palmitoylation and surface expression, reduces endosomal recycling, and promotes lysosomal degradation—providing a pharmacological prototype for site-level antagonism 25. B7-H4 also depends on ZDHHC3-catalyzed palmitoylation at Cys130 to avoid lysosomal degradation and sustain its immunosuppressive effect, indicating a multi-checkpoint lipidation equilibrium that maintains immune suppression 154. Within the innate-immunity axis, inhibition of LYPLAL1-mediated cGAS depalmitoylation increases cGAS lipidation and enhances type I interferon (IFN-I) signaling, which synergistically augments PD-1 blockade efficacy in vivo. This defines a manipulable “depalmitoylation-lipidation homeostasis-IFN-I” regulatory axis 109. Similarly, CPT1A induction following epigenetic perturbation recruits ER-resident ZDHHC4 to the mitochondrial vicinity, promoting MAVS Cys79 palmitoylation and stabilization, thereby amplifying IFN-I activation and functionally synergizing with PD-1 blockade 155. At the immune-vascular interface, endothelial STING-JAK1 interaction promotes vascular normalization and CD8⁺ T-cell infiltration. Given that STING activation strictly depends on Golgi-localized palmitoylation at Cys88/Cys91, its lipidation state likely constrains this vascular-immune cross-talk 156, 157. Extending to organotropism and metastasis, ZDHHC20 remodels the tumor-cell membrane proteome and reduces NK-cell recognition, thereby facilitating distant metastasis in pancreatic cancer—solidifying the causal chain of “palmitoylation → immune evasion → metastatic colonization.” 127

Within innate immune and inflammatory-sensing networks, S-palmitoylation functions both as a licensing step for signal initiation and a rheostat that calibrates signal amplitude. For example, STING palmitoylation at Cys88 and Cys91 on the Golgi is indispensable for TBK1/IRF3 recruitment and the amplification of type I interferon (IFN-I) signaling. This lipidation also induces cholesterol-dependent membrane ordering and STING clustering, enabling the sequential process of “localization → assembly → amplification.” 157, 158 In the inflammasome axis, ZDHHC5-mediated NLRP3 palmitoylation strengthens its interaction with NEK7 and promotes inflammasome assembly, whereas the depalmitoylase ABHD17A acts as a counterbalancing brake. Conversely, ZDHHC12-mediated NLRP3 palmitoylation can trigger chaperone-mediated autophagy (CMA), thereby limiting chronic inflammation—together forming a bidirectional “on-off” regulatory loop 108, 159. At the metabolism-immunity interface, the ZDHHC3-SCAP palmitoylation axis disrupts cholesterol homeostatic feedback, suppresses CD4⁺ T-cell effector functions, and promotes immune evasion in hepatocellular carcinoma (HCC). Inhibition of this PAT, when combined with PD-1 blockade, produces synergistic antitumor effects in mouse models 73. On the depalmitoylation side, targeting LYPLAL1 to prevent cGAS depalmitoylation enhances its enzymatic activity and IFN-I response, thereby improving immune-checkpoint blockade (ICB) efficacy. Intriguingly, ZDHHC18-mediated palmitoylation of cGAS at Cys474 exerts the opposite effect by suppressing cGAS dimerization and activity, illustrating a tug-of-war control between PATs and depalmitoylases 109, 160. Expanding this logic, MAVS palmitoylation serves as an aggregation switch for innate antiviral and antitumor immunity, predominantly catalyzed by ZDHHC12 (with ZDHHC4 contributing in certain melanoma contexts), directly coupling RIG-I-like receptor activation to downstream immune signaling 161. At the tissue level, endothelial STING-JAK1 interactions, modulated by STING's palmitoylation status, drive vascular normalization and CD8⁺ T-cell infiltration, thereby amplifying systemic antitumor immunity 156. Comprehensive reviews have now consolidated the palmitoylation-regulatory landscape of PRRs (e.g., cGAS-STING, NLRP3) and identified multiple druggable nodes, providing a mechanistic rationale for “palmitoylation correction + ICB/metabolic co-therapy” as a generalizable immunotherapeutic framework 162, 163.

Within the antioxidant and ferroptosis axis, the balance between palmitoylation and depalmitoylation serves as a site-level molecular lever that sets the immune-sensitivity threshold, directly influencing tumor-cell survival and immune clearance 83. Targeting the depalmitoylase PPT1 increases Gpx1 S-palmitoylation, restores its peroxidase activity, and suppresses pathological angiogenesis—demonstrating the feasibility of a “depalmitoylation-to-repalmitoylation” reverse-intervention strategy 133. At the ferroptosis checkpoint, GPX4 S-palmitoylation, catalyzed by the druggable enzyme ZDHHC8, determines cellular resistance to ferroptosis and susceptibility to CD8⁺ T cell-mediated killing. Inhibiting this site simultaneously enhances antitumor immunity and suppresses tumor growth 83. Independent studies have shown that GPX4 also undergoes reversible palmitoylation at Cys66, catalyzed by ZDHHC20 and removed by APT2. Disrupting this site—or applying broad-spectrum palmitoylation blockade—significantly increases ferroptotic sensitivity, reinforcing the “palmitoylation-ferroptosis” universal mechanism 84. At the intersection of innate immunity, inhibition of LYPLAL1-mediated cGAS depalmitoylation boosts cGAS activation, enhances IFN-I signaling, and improves immune-checkpoint blockade (ICB) responsiveness—validating the “depalmitoylation-axis sensitization” concept 109. Furthermore, CPT1A upregulation, driven by epigenetic perturbation, can recruit ZDHHC4 to the mitochondrial outer membrane, promoting MAVS palmitoylation and stabilization, which amplifies type I interferon responses and strengthens antitumor immunity 155. At the systemic level, activation of the endothelial STING-JAK1 hub depends on STING palmitoylation at Golgi Cys88/91, a modification that supports vascular normalization and CD8⁺ T cell infiltration 156 while providing a structural basis for the optimization of STING agonists 157. Consistently, engineered STING-activating nanoparticles have demonstrated synergistic effects with immunotherapy across multiple tumor models by normalizing vasculature, enhancing T-cell infiltration, and reinforcing checkpoint efficacy 164.

Importantly, the immune consequences of palmitoylation are not restricted to tumor-cell-intrinsic checkpoint stabilization. A broader non-cell-autonomous dimension is already emerging across the tumor microenvironment 165. This non-cell-autonomous perspective is currently best supported in three compartments—vascular endothelium, myeloid cells, and metastatic immune surveillance—whereas direct palmitoylation-level evidence in cancer-associated fibroblasts (CAFs) remains comparatively limited 165, 166. In the vascular compartment, endothelial STING signaling promotes vessel normalization and CD8⁺ T-cell infiltration 156, and this pathway is mechanistically gated by Golgi-dependent STING palmitoylation 157. At the metastatic interface, ZDHHC20-dependent remodeling of the tumor-cell surface proteome reduces NK-cell-mediated recognition during pancreatic-cancer colonization, thereby linking palmitoylation to organ-specific immune escape 127. In the myeloid compartment, PPT1⁺ macrophages define an immunosuppressive contexture and are associated with inferior immunotherapy responsiveness in hepatocellular carcinoma 38. By contrast, in CAFs and other stromal fibroblast populations, current evidence primarily supports their broad importance as tumor-microenvironmental regulators rather than a defined ZDHHC-substrate circuit; therefore, CAF-related palmitoylation should be framed as an important but still underdeveloped area requiring single-cell, spatial, and cell-type-resolved palmitoyl-proteomic validation 165, 166. Together, these observations indicate that palmitoylation-dependent regulation extends across tumor, immune, vascular, and stromal compartments rather than remaining confined to malignant cells alone 38, 127, 156, 157, 165, 166. However, direct mechanistic evidence is still concentrated in a limited set of endothelial, metastatic-colonization, and myeloid examples—most notably endothelial STING-dependent vascular normalization, which is gated by STING palmitoylation 156, 157, ZDHHC20-linked regulation of NK-cell-dependent metastatic outgrowth in pancreatic cancer 127, and PPT1-positive macrophage states in hepatocellular carcinoma 38—whereas the broader CAF/stromal compartment remains largely inferential at present 165, 166. Accordingly, the broader microenvironmental model should still be regarded as suggestive rather than fully cell-type-resolved.

Collectively, these findings establish “site-specific palmitoylation → membrane stabilization / complex assembly → signal persistence” as a unifying mechanistic scaffold for immune evasion, forming a pharmacologically actionable cause-effect cascade 162. At the checkpoint level, PD-L1 palmitoylation enhances its plasma-membrane stability and prevents lysosomal degradation, thereby amplifying inhibitory ligand output 27. PD-1 palmitoylation represents a critical threshold for its expression and suppressive function and can be antagonized by site-competitive peptides or small molecules 25. Likewise, ZDHHC9-catalyzed palmitoylation of TIM-3 prevents HRD1-mediated ubiquitination, stabilizing the receptor at the membrane and driving an exhaustion phenotype 106. Within innate immune signaling, STING palmitoylation at Golgi Cys88/91 acts as a licensing step for TBK1/IRF3 recruitment and IFN-I activation. This modification can be blocked by covalent palmitoylation inhibitors, demonstrating a “localization → assembly → amplification” gating mechanism 107, 157. Meanwhile, the palmitoylation-depalmitoylation balance of cGAS governs its dimerization and signaling strength; LYPLAL1 inhibition enhances cGAS activation and potentiates the response to PD-1 blockade 109. Similarly, NLRP3 palmitoylation facilitates NEK7 binding and inflammasome assembly, while depalmitoylation provides a dynamic brake on activation 108. In the metabolism-immunity interface, SCAP palmitoylation activates SREBP, disrupts cholesterol homeostasis, and promotes HCC immune escape, while co-targeting this axis with PAT inhibition plus PD-1 blockade produces synergistic tumor suppression 73. The GPX lipidation axis tightly couples the ferroptosis threshold to immune sensitivity: ZDHHC8-mediated GPX4 palmitoylation stabilizes the enzyme and reduces susceptibility to ferroptosis and CD8⁺ T-cell killing 83, whereas inhibition of the depalmitoylase PPT1 enhances Gpx1 lipidation and suppresses pathological angiogenesis, providing a tangible handle for immune-metabolic intervention 133. Accordingly, PAT inhibition and site-specific blockade offer direct mechanistic leverage. For instance, inhibiting DHHC3—or correcting dysfunction within the ZDHHC3-SCAP node—destabilizes PD-L1, reverses cholesterol-driven immune suppression, and synergizes with anti-PD-1 therapy 28, 73. Targeting the ZDHHC8-GPX4 axis enhances both ferroptosis and antitumor immunity 83; inhibiting depalmitoylases such as PPT1 or LYPLAL1 resets oxidative and innate-immune thresholds via “depalmitoylation-to-re-palmitoylation” modulation 109, 133; and PD-1 lipidation antagonists represent a site-resolved, druggable paradigm for peptide or small-molecule design 25. At the systems level, these lipidation-based interventions also complement pathways that amplify IFN-I signaling. Epigenetically induced CPT1A upregulation recruits ZDHHC4 to the mitochondrial outer membrane, promoting MAVS palmitoylation and stabilization, which synergizes with PD-1 blockade to enhance antitumor efficacy 155. In parallel, activation of the endothelial STING-JAK1 hub, regulated by STING palmitoylation, promotes vascular normalization and CD8⁺ T-cell infiltration, reinforcing a tissue-scale “lipidation network × immunotherapy” convergence 156.

## 5. The Depalmitoylation System and Dynamic ZDHHC Equilibrium: A Precision Switch for Tumor Signaling

Depalmitoylation constitutes the counter-regulatory arm of the palmitoylation cycle and is mediated mainly by APT1/2, ABHD17 family members, and lysosomal thioesterases such as PPT1 29, 49, 50, 94. Rather than merely reversing ZDHHC activity, these enzymes determine substrate residence time, membrane recycling, and signaling duration, thereby functioning as dynamic brakes on forward lipid flux. Mechanistically, APT2 associates with membranes through electrostatic attraction and insertion of its β-tongue loop, while S-acylation promotes membrane residence and stabilization; once membrane bound, APT2 extracts substrate acyl chains into a hydrophobic pocket for hydrolysis 49, 167, 168. In cancer models, this reverse arm is pharmacologically relevant: ABHD17 inhibition perturbs N-Ras recycling and attenuates oncogenic signaling 50, 94, APT2 inhibition restores Scribble palmitoylation and membrane localization in EMT models 122, and PPT1 inhibitors such as DC661 and GNS561 show preclinical and early clinical activity while enhancing the response to PD-1 blockade 23, 24, 169, 170. Against this background, the following section discusses how depalmitoylases and ZDHHC enzymes together establish a dynamic equilibrium that is both mechanistically informative and therapeutically tractable.

The Ras palmitoylation cycle exemplifies the paradigm of “dynamic localization equals signal tuning.” Both H-Ras and N-Ras undergo continuous palmitoylation-depalmitoylation shuttling between the Golgi apparatus, plasma membrane, and cytosolic pools. Disruption of this cycle causes Ras mislocalization and a reduction in downstream signal amplitude—most notably attenuated ERK activation 171. At the catalytic level, ZDHHC9, together with its cofactor GCP16, forms the core palmitoyltransferase complex responsible for H-/N-Ras modification, defining the enzymatic source of Ras lipidation 46. GCP16 serves as an exclusive stabilizing cofactor that maintains ZDHHC9 subfamily integrity and constrains its subcellular localization 47. Recent cryo-EM analyses further resolved the fine architecture of the DHHC9-GCP16 complex, revealing a coupled mechanism of “auto-palmitoylation → conformational tuning → substrate selectivity.” These findings provide direct structural support for the structure-guided design of DHHC9 inhibitors 172. On the depalmitoylation arm, ABHD17A/B/C have been identified as the major N-Ras depalmitoylases, governing the rate and extent of N-Ras retrograde trafficking from the plasma membrane back to the cytosolic and endomembrane compartments 50. The selective covalent inhibitor ABD957, which targets the ABHD17 family, effectively blocks depalmitoylation, decreases Ras membrane localization, and suppresses proliferation in NRAS-dependent tumor models, validating the ABHD17-Ras axis as a pharmacologically vulnerable node 94. By contrast, the dual APT1/2 inhibitor Palmostatin B disrupts Ras localization but lacks selectivity and exhibits widespread proteomic off-target effects 54; 2-bromopalmitate (2-BP) is even broader in scope, inhibiting both DHHC and depalmitoylase families (including APT1/2), and thus should not be used as sole evidence for lipidation dependency 21, 53. At the transport-lipidation interface, RAB27B cooperates with ZDHHC9 to enhance NRAS palmitoylation and direct its trafficking to the plasma membrane, forming a “transport → lipidation → signaling” feed-forward loop. This coupling underscores the plasticity and druggability of the Ras lipidation cycle in cancer contexts 93. Moreover, the mono- versus di-palmitoylation states of Ras isoforms determine their Golgi residency and membrane microdomain preference. The rhythmic balance of depalmitoylation and re-palmitoylation defines the temporal window for Golgi/ER recycling and re-export to the plasma membrane, thereby functioning as a molecular limiter of pathway output 55, 57, 173-175. The tripartite coupling of DHHC9-GCP16 catalysis, ABHD17-mediated depalmitoylation, and small-GTPase trafficking completes a mechanistic loop linking site-specific palmitoylation to membrane anchoring, complex assembly, and signaling amplitude. This framework provides a testable basis for combinations such as ABHD17 inhibition plus MAPK blockade or selective DHHC9 inhibition in NRAS-driven cancers 94, 172, 176.

At the interface of programmed cell death and metabolic susceptibility, the ferroptosis threshold of tumor cells is finely tuned by reversible S-palmitoylation of GPX4. Two independent studies identified ZDHHC8 and ZDHHC20 as catalytic enzymes that palmitoylate GPX4, thereby enhancing its stability, reducing lipid peroxidation, and suppressing ferroptosis 83, 84. Specifically, ZDHHC20 lipidates Cys66 of GPX4, while the depalmitoylase APT2 removes this modification, maintaining a dynamic “palmitoylation-depalmitoylation” equilibrium that directly links site modification to ferroptosis sensitivity 49, 84. Functionally, selective inhibition of the ZDHHC8-GPX4 axis significantly increases ferroptosis and CD8⁺ T-cell-mediated cytotoxicity across multiple tumor models, producing reproducible synergy with immune-checkpoint blockade (ICB) 83. This “threshold lever” is not isolated but connected to an upstream antioxidant network. AMPKα1-mediated phosphorylation of ZDHHC8 promotes SLC7A11 palmitoylation and plasma-membrane stabilization, enhancing resistance to ferroptosis through an independent pathway—illustrating the plastic coupling between lipidation and the antioxidant defense system 132. Consistently, the long noncoding RNA DUXAP8 sustains SLC7A11 lipidation and lysosomal evasion, elevating the antioxidant threshold and dampening sorafenib-induced ferroptosis 131. The depalmitoylation side of this circuit also offers druggable entry points. PPT1, a lysosomal depalmitoylase, has been identified as the direct molecular target of chloroquine, hydroxychloroquine, and the dimeric derivative DC661. PPT1 inhibition produces dual outcomes: 1 lysosomal deacidification and autophagy blockade, leading to metabolic stress and cytotoxicity; and 2 strong synergy with anti-PD-1 therapy in melanoma and other models 23, 169. Clinically, the oral PPT1 inhibitor GNS561 (Ezurpimtrostat) has completed early-phase trials, demonstrating favorable pharmacokinetics and tolerability 24, 26. In hepatocellular carcinoma (HCC), GNS561 combined with PD-1 blockade remodels the immune microenvironment and enhances CD8⁺ T-cell infiltration, validating the “depalmitoylation sensitization × ICB” therapeutic paradigm 170. Histologically, PPT1⁺ myeloid cells correlate with immunosuppressive contexture and poor prognosis in HCC specimens, marking the “PPT1-myeloid phenotype” as a clinically relevant immunomodulatory target 38. At the redox-angiogenesis interface, the PPT1-Gpx1 axis couples depalmitoylation-repalmitoylation balance to vascular remodeling: inhibiting PPT1 paradoxically enhances Gpx1 lipidation and peroxidase activity, thereby downregulating the ROS-HIF-1α-VEGF cascade and suppressing pathological angiogenesis 133. Together, the ZDHHC8/20-GPX4 (stabilization and ferroptosis suppression) and PPT1-Gpx1 (vascular-redox coupling) axes converge on a “site-level druggable hub” that integrates ferroptosis, vascular homeostasis, and antitumor immunity. When the therapeutic goal is to promote ferroptosis or immune activation, inhibition of the GPX4 lipidation axis or destabilization of the protein is preferred 83, 84; conversely, in anti-angiogenic contexts, PPT1 inhibition enhances Gpx1 lipidation and antioxidant defense 23, 169. From a translational standpoint, we advocate a three-tier validation chain—site, enzyme, function (i.e., site-directed mutation, defined catalytic component, and ferroptosis/immune readout)—rather than single-drug inference. Integrating this with high-throughput mechanistic tools enables accurate quantification of lipidation-delipidation dynamics and ensures reproducibility and mechanistic fidelity 35, 40, 49, 51. On a broader scale, systematic reviews and expert guidelines have formalized the biological and methodological framework of “ferroptosis × immunotherapy”, supporting these strategies as cross-cancer, generalizable interventions 177, 178.

The innate immune axis is exquisitely sensitive to lipidation status. STING S-palmitoylation at Cys88 and Cys91 on the Golgi serves as a licensing step for its activation and for TBK1/IRF3 recruitment; either 2-bromopalmitate treatment or site-directed mutation at these residues markedly suppresses type I interferon (IFN-I) output 157. Single-molecule localization microscopy further demonstrates that STING can form dense clusters at the trans-Golgi network (TGN) only after palmitoylation—providing the physical basis for signal amplification 158. Complementarily, the depalmitoylation of cGAS is tightly controlled by the depalmitoylase LYPLAL1. Pharmacologic or genetic inhibition of LYPLAL1 enhances cGAS-DNA binding and the IFN-I response, significantly improving anti-PD-1 efficacy in mouse models 109. Conversely, ZDHHC18 negatively regulates innate immunity by promoting site-specific palmitoylation of cGAS; deletion or inhibition of ZDHHC18 augments DNA sensing and antitumor response 160. From a pharmacologic perspective, 4-octyl itaconate (4-OI) directly alkylates STING Cys91, blocking its palmitoylation and oligomerization. Similarly, endogenous nitro-fatty acids suppress STING palmitoylation and IFN-I release through nitro-alkylation, demonstrating that “anti-lipidation” represents an effective downregulatory mechanism 107, 179. In line with this, covalent small molecules such as H-151 and C-176 occupy Cys91, thereby preventing STING palmitoylation at the Golgi, disrupting oligomer assembly, and suppressing TBK1-IRF3 activation 180, 181. Lipid metabolic flux also shapes this licensing step. For instance, fatty acid synthase (FASN) influences STING palmitoylation and signaling amplitude by modulating the substrate pool 182, while the lipid peroxidation product 4-hydroxynonenal (4-HNE) antagonizes this process through protein carbonylation, attenuating STING-driven pathway amplification 183. Collectively, “STING site-specific palmitoylation licensing” and “cGAS depalmitoylation release” together define the sensitivity window of the antitumor innate-immune “antenna.” This dual-site regulation provides a mechanistic foundation for “site-specific intervention + checkpoint blockade” synergy—a conceptual framework now reinforced by multiple high-quality systematic reviews 184.

At the molecular level of the depalmitoylase arm, membrane association and catalysis by APT2 proceed through a highly ordered four-step sequence. First, the enzyme's positively charged surface electrostatically attracts the negatively charged lipid bilayer. Second, the β-tongue hydrophobic loop inserts into the bilayer to achieve initial anchoring. Third, transient self-S-acylation of its N-terminal cysteine, mediated by ZDHHC3/7, stabilizes membrane residency. Finally, the enzyme's hydrophobic pocket extracts, captures, and positions the substrate's acyl chain for hydrolysis—completing the depalmitoylation cycle 49, 185. This sequence integrates electrostatic, hydrophobic, and covalent selection layers, defining the physicochemical essence of depalmitoylation and explaining the substrate selectivity of APT2, which depends on both lipid-chain recognition and adjacent sequence or conformational cues 29. Despite their shared catalytic fold, APT1 and APT2 differ in active-site geometry and ligand entry pathways. Co-crystal structures of their selective inhibitors—ML348 (APT1-biased) and ML349 (APT2-biased)—reveal distinct “gatekeeper residues” within the β5-α2 loop and hydrophobic tunnel. These differences orient inhibitor and lipid binding trajectories nearly orthogonally, providing the structural rationale for isoform-specific pharmacology and functional divergence 168, 186. Cell-based evidence further confirms that selective inhibition of APT2 specifically restores membrane localization and S-palmitoylation of the polarity protein Scribble while suppressing MAPK activity, identifying APT2 as a pathogenic driver of EMT-associated signaling 122. Substrate recognition, however, extends beyond lipid moieties: amino acids distal to the palmitoylated cysteine modulate APT1/2 specificity 187. Early chemical biology also established Palmostatin B/M as dual APT1/2 cellular targets, laying the foundation for subsequent selective probes and pharmacologic optimization 22. Depalmitoylases may also fulfill non-catalytic, structural roles. In fission yeast, the palmitoyl hydrolase family member Phi1 bridges cohesin and RSC (Brg1 homolog) complexes to promote chromosome loading. The same study identified APT1/2-RAD21/BRG1 interactions in human cells as catalysis-independent, with nuclear APT1 accumulation correlating with poor clinical outcomes—highlighting a “non-enzymatic dimension” of biological impact 188. This suggests that inhibiting enzymatic activity alone may not fully capture the pathophysiologic scope of the depalmitoylation system. A more robust strategy may involve “dual-site targeting”: one arm aimed at the catalytic pocket or lipid channel (as exemplified by ML348/ML349) 168, and the other at scaffold or bridging interfaces (guided by Phi1/human APT1 interaction models), to achieve a broader and more selective therapeutic window 188. Notably, APT membrane residency and stability themselves are finely tuned by their own S-acylation cycles and β-tongue anchoring, underscoring that drug design should account not only for active-site chemistry but also for membrane affinity and self-regulatory dynamics to achieve spatiotemporal precision 29, 49, 167, 185. From a systems perspective, localization and activity of membrane-associated signaling proteins such as Ras are continuously maintained by the ZDHHC-APT palmitoylation-depalmitoylation cycle. This dynamic coupling between the “addition” and “removal” arms mechanistically supports the feasibility—and necessity—of synchronous dual-pathway intervention 29, 176, 189. These insights, rooted in the earliest biochemical characterization of APT enzymes and Ras acylation-deacylation cycling, provide the structural and functional foundation for advancing the field's chemical biology and therapeutic development.

At the population and phenotypic level, integrated multi-omic and clinical cohort analyses reveal a consistent association between the palmitoylation/depalmitoylation balance and immune escape. In lung adenocarcinoma (LUAD), high expression of ZDHHC4/12/18/24 and the depalmitoylase APT2 correlates with reduced CD8⁺ T-cell infiltration, shortened overall survival, and upregulation of the immune checkpoint CD276 (B7-H3), defining a population-level anchor that links the lipidation axis to an immunosuppressive tumor microenvironment 59. At the cellular and functional level, upregulation of APT1/LYPLA1 in non-small cell lung cancer (NSCLC) promotes migration and epithelial-mesenchymal transition (EMT), while silencing LYPLA1 reverses this phenotype by restoring E-cadherin and downregulating N-cadherin, Vimentin, and Snail, thereby strengthening the functional connection between depalmitoylation and loss of polarity/invasiveness 190. The checkpoint lipidation loop is now being pharmacologically closed. Inhibition of PD-L1 S-palmitoylation triggers lysosomal degradation and enhances T-cell cytotoxicity, a result that has been repeatedly validated across tumor models using both engineered inhibitory peptides and chemical inhibitors 27, 82. At the enzyme level, the marine natural product Benzosceptrin C directly targets DHHC3, induces PD-L1 degradation, and synergizes with CTLA-4 blockade, validating the “DHHC3→PD-L1” enzyme-site axis as a feasible point of therapeutic intervention 28. In parallel, a degradation-based strategy targeting DHHC3 (cp-PCCs) has demonstrated efficient PD-L1 downregulation and immune reactivation in checkpoint-refractory models, closing the pharmacodynamic loop of PD-L1 inhibition 191. At the metabolism-immunity interface, ZDHHC3-mediated SCAP S-acylation disrupts cholesterol-synthesis feedback, reprograms cholesterol metabolism, and promotes immune evasion in hepatocellular carcinoma (HCC), forming a direct molecular bridge among lipidation, metabolism, and immune phenotype 73. Beyond PD-L1, TIM-3 S-palmitoylation, catalyzed by DHHC9, enhances receptor stability and drives immune exhaustion, illustrating a multi-checkpoint lipidation equilibrium that reinforces immunosuppression 106. These population-level, molecular, and pharmacologic observations support a unified spatiotemporal model in which palmitoylation functions as a licensing step and depalmitoylation acts as a rheostat. Together with the ZDHHC-APT cycle that governs Ras and other peripheral membrane signaling proteins 8, 11, this dual control intersects with cGAS-STING signaling at the level of site-specific lipidation 109, 157, 160 and reshapes the ferroptosis threshold through regulators such as GPX4 83, 84. Systematic perturbation of the Ras palmitoylation-depalmitoylation cycle has shown selective suppression in NRAS-mutant models, providing a functional precedent for site-specific correction combined with immuno-metabolic and targeted therapies 192.

## 6. Therapeutic Frontiers in Targeting the ZDHHC Family and Protein Palmitoylation: Precision Intervention Strategies and Emerging Challenges

**Figure 3:**
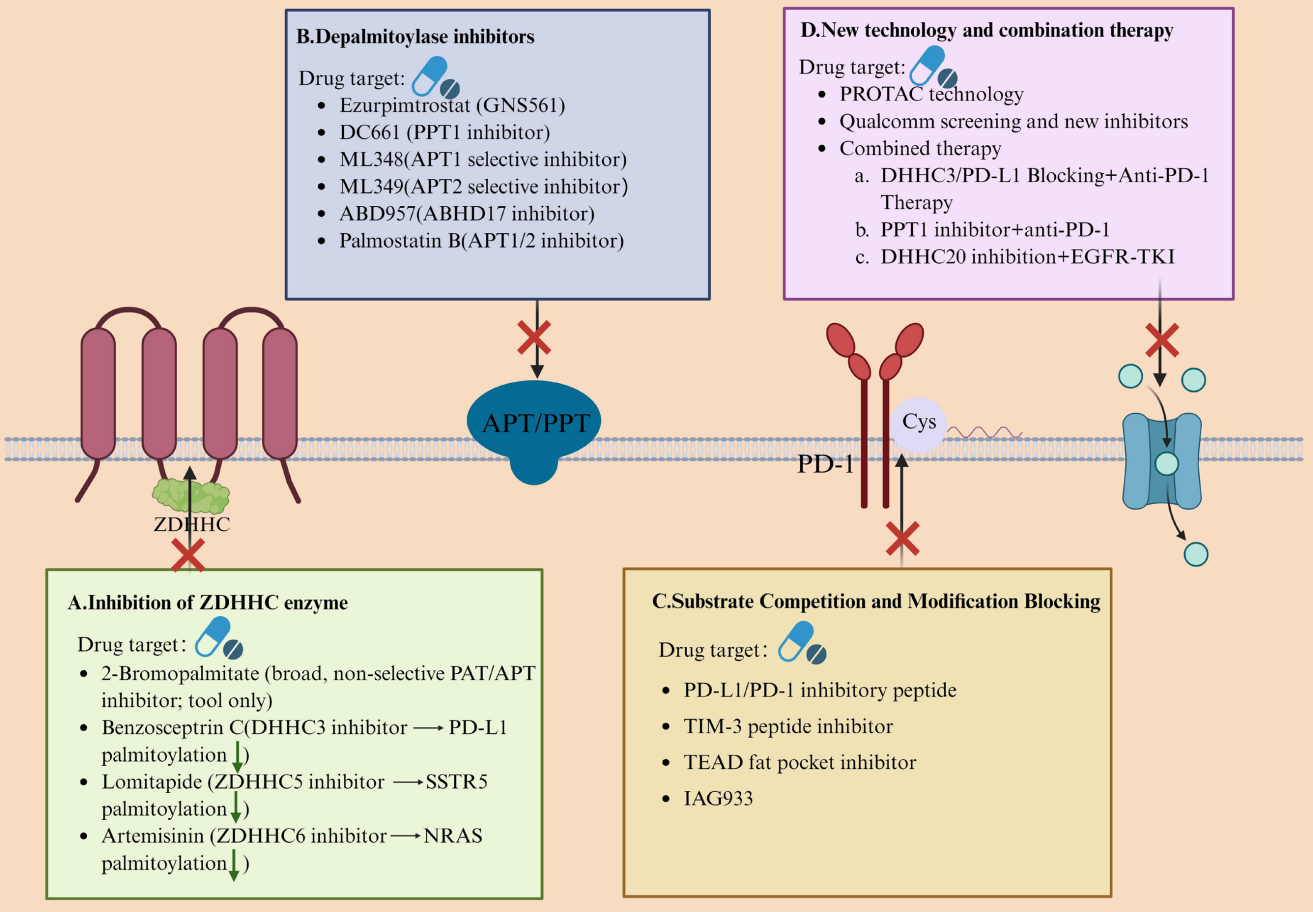
** Therapeutic strategies targeting the ZDHHC-palmitoylation axis in cancer.** Conceptual summary of druggable entry points along the palmitoylation-depalmitoylation cycle and their integration with immune and targeted therapies. A, Inhibition of ZDHHC palmitoyltransferases. Small-molecule and repurposed agents directly inhibit ZDHHC enzymes to reduce substrate S-palmitoylation, exemplified by broad-spectrum tool compounds such as 2-bromopalmitate, and more selective or repurposed inhibitors that target DHHC3 (e.g. Benzosceptrin C, leading to PD-L1 destabilisation), ZDHHC5 (Lomitapide, impairing SSTR5 palmitoylation) and ZDHHC6 (artemisinin, reducing NRAS palmitoylation). B, Depalmitoylase inhibitors. Pharmacological blockade of depalmitoylases stabilises palmitoylated substrates or perturbs palmitoylation cycles, as illustrated by PPT1 inhibitors Ezurpimtrostat (GNS561) and DC661, isoform-selective APT1/2 inhibitors (ML348, ML349), the ABHD17 inhibitor ABD957 and the dual APT1/2 tool compound Palmostatin B. C, Substrate competition and site-directed modification blocking. Peptidic and small-molecule antagonists interfere directly with palmitoylation of key substrates, including PD-L1/PD-1 and TIM-3 inhibitory peptides, as well as TEAD lipid-pocket inhibitors and the YAP-TEAD interface blocker IAG933, thereby destabilising checkpoints or transcriptional effectors. D, New technologies and combination regimens. Emerging approaches encompass targeted protein degradation of DHHC enzymes (e.g. PROTAC-like chimeras against DHHC3), high-throughput screening platforms for next-generation PAT and depalmitoylase inhibitors, and mechanism-based combinations such as DHHC3/PD-L1 blockade plus anti-PD-1, PPT1 inhibition plus anti-PD-1, or DHHC20 inhibition plus EGFR tyrosine-kinase inhibitors to synergistically enhance antitumor immunity and targeted-therapy efficacy.

Therapeutic manipulation of the reversible palmitoylation-depalmitoylation axis can be broadly organised into three complementary, but unevenly mature, strategies: direct enzyme targeting, encompassing structurally guided inhibition of ZDHHC enzymes enabled by recent structural and screening platforms 32-34 and pharmacological suppression of depalmitoylases such as PPT1, ABHD17, and APT1/2 23, 24, 49, 50, 94; substrate-site intervention, which blocks S-palmitoylation or exposes destabilising residues on key substrates to modulate protein turnover and signaling amplitude 25, 27, 28; and upstream lipidation blockade, in which inhibition of membrane-bound O-acyltransferases (MBOATs) such as PORCN and HHAT disrupts Wnt or Hedgehog ligand lipidation and secretion 193, 194. At present, these strategies should be regarded as translational hypotheses with heterogeneous evidentiary maturity rather than as a clinically validated therapeutic class 29, 35, 37. Together, these three tiers delineate complementary intervention nodes along the palmitoylation cycle and its key substrates, but they should be viewed as a conceptual therapeutic framework rather than a clinically mature one, because several prerequisites for clinical deployment remain unmet: isoform-selective inhibition with adequate pharmacological properties despite recent structural and screening progress 32-34, 195, discrimination between catalytic and non-catalytic/scaffolding functions 188, substrate-level pharmacodynamic confirmation and target-engagement read-outs based on enzyme-specific or site-resolved S-acylation assays 29, 35, 37, 39, 40, and biomarker-guided enrichment of tumor contexts that are demonstrably dependent on the relevant palmitoylation circuit, a requirement that remains insufficiently established in current early clinical and biomarker studies 24, 38, 59 **(Figure 3)**. Representative agents that operationalize these tiers—including clinical-stage PPT1 inhibitors, ABHD17 and DHHC3 modulators, and PORCN/HHAT blockers, together with their mechanisms, developmental stage, and preferred combination partners—are summarized in **(Table 3)**. At the enzyme level, crystallographic studies of ZDHHC family members have elucidated the structural basis of acyl-chain recognition by their transmembrane hydrophobic cavity and cytoplasmic catalytic core, providing clear structure-activity anchors for structure-guided, site-selective small-molecule design 32. On the depalmitoylase side, the ABHD17 family has yielded a selective irreversible inhibitor, ABD957, which suppresses depalmitoylation and attenuates oncogenic signaling in NRAS-dependent models, highlighting the subtype-specific druggability of the “ABHD17-Ras” axis 94. By contrast, classic tool compounds such as 2-bromopalmitate (2-BP) and Palmostatin B alter protein localization and signaling but display substantial off-target activity—2-BP directly inhibits APT1/2, while Palmostatin B broadly affects the proteome. Consequently, study design should avoid relying solely on single-agent phenomenology and instead establish a three-tier evidence chain—site, enzyme, pathway to ensure mechanistic validity 53, 54. Clinically, among depalmitoylase-directed strategies, PPT1 is currently the most clinically advanced node, with preclinical target validation for PPT1 inhibition 23, 26 and first-in-human evaluation of GNS561/Ezurpimtrostat 24. The dimeric chloroquine derivative DC661, originally linked to PPT1 targeting in cancer 23, enhances sorafenib sensitivity in hepatocellular carcinoma models 196; PPT1 inhibition has also been shown to enhance anti-PD-1 activity in melanoma models 169. The oral PPT1 inhibitor GNS561 (Ezurpimtrostat) has completed first-in-human evaluation with acceptable safety, marked hepatic exposure, and disease-stabilization signals rather than established objective-response or biomarker-guided efficacy 24. Accordingly, the current PPT1 experience should be interpreted as evidence of clinical exposure and target-class tractability, not as proof that depalmitoylase inhibition has achieved clinically validated antitumor efficacy 24. Available clinical data do not yet establish durable benefit, optimal patient selection, a predictive biomarker, or a substrate-level pharmacodynamic read-out in patient specimens 24. Preclinical and translational studies support continued combination-based evaluation, particularly with immune-checkpoint blockade, but these studies remain hypothesis-generating until prospective biomarker-linked clinical benefit is demonstrated 26, 169, 170. More generally, pathway-level or biomarker read-outs—including VEGF-related angiogenic suppression, immune-checkpoint destabilization, CD8⁺ T-cell infiltration, or lysosomal/autophagy modulation—should be interpreted as pharmacodynamic evidence rather than as proof of clinical efficacy. Such read-outs can support mechanistic plausibility and target engagement, but they do not substitute for clinically established endpoints such as objective response, progression-free survival, overall survival, durable benefit, or prospectively validated biomarker-defined patient selection 133, 197, 198. More broadly, progress in palmitoylation-directed therapy will require more robust target-engagement and substrate-level pharmacodynamic tools 35, 195, clearer definition of therapeutic windows for DHHC-directed compounds 24, 195, strategies to disentangle isozyme/substrate redundancy 35, 199, and close attention to adaptive rewiring under chronic target suppression 200. Drug repurposing has also produced early leads on the PAT side: Lomitapide, an FDA-approved lipid-lowering agent, was recently identified as a ZDHHC5 inhibitor that blocks SSTR5 palmitoylation and suppresses pancreatic-cancer proliferation, demonstrating the feasibility of repositioning approved molecules for DHHC inhibition 201. At the substrate-level correction tier, inhibition of PD-L1 palmitoylation promotes lysosomal degradation and enhances T-cell cytotoxicity, establishing a canonical paradigm of “site blockade → destabilization → immune sensitization” 27. Similarly, Benzosceptrin C, a marine natural product, directly targets DHHC3 to induce PD-L1 degradation and synergize with CTLA-4 blockade, offering an enzyme-substrate dual-node framework for rational combination therapy 28. Within the metabolism-immunity coupling domain, ZDHHC3-mediated SCAP S-acylation sustains cholesterol synthesis and drives immune evasion in HCC; inhibition of this axis achieves tri-modal coordination of “lipidation-metabolism-immunity.” 73 Upstream of these pathways, blockade of lipid-dependent ligand secretion is emerging as a complementary strategy. The PORCN inhibitor WNT974 (LGK974) has completed single-agent phase I testing and has also been evaluated in a phase Ib/II combination with encorafenib plus cetuximab in BRAF V600E-mutant metastatic colorectal cancer 193, 202, whereas ETC-159 provides preclinical proof-of-concept for ligand-lipidation blockade in genetically defined Wnt-addicted models 203. These data position PORCN inhibition as a clinical comparator for upstream Wnt-ligand lipidation blockade rather than as direct validation of ZDHHC-targeted therapy, and they also show that pathway druggability requires tumor-context selection before broad clinical efficacy can be inferred 193, 202, 203. In the Hedgehog pathway, HHAT inhibitors such as RU-SKI-43 and IMP-1575 support pharmacologic blockade of Hedgehog ligand palmitoylation at the probe/lead stage, but remain preclinical and require further optimization for selectivity and in vivo pharmacology 194, 204, 205.

At the substrate-site and upstream ligand-lipidation levels, we retain representative therapeutic archetypes rather than re-expanding mechanistic details already developed in Sections 4.4 and 5. Beyond isolated compound-by-compound effects, selected natural-product-derived or repurposed small molecules discussed in this review illustrate a convergent, multi-node pharmacologic logic within the palmitoylation network. Curcumin attenuates integrin β4 palmitoylation while inhibiting ZDHHC3 autoacylation at an adhesion/invasion interface 111; artemisinin covalently inhibits ZDHHC6 and suppresses NRAS palmitoylation with downstream ERK/AKT attenuation 92; genistein disrupts the ZDHHC17-MAP2K4 signaling interface in glioblastoma 87; and Benzosceptrin C targets DHHC3 to destabilize PD-L1 and enhance antitumor immunity 28. These examples suggest that multi-node modulation may be useful when several palmitoylation-dependent modules converge on compensatory pathway persistence, immune-checkpoint stability, or invasion; however, such pleiotropy should be interpreted as a therapeutic hypothesis rather than an intrinsic pharmacologic advantage unless supported by site-, enzyme-, pathway-, and phenotype-level validation 29, 35, 40. Checkpoint lipidation blockade is summarized through PD-1 25, PD-L1 27, 28, TIM-3 106, and B7-H4 154 as examples of site-directed control of checkpoint expression, membrane stability, receptor turnover, or exhaustion signaling. ZDHHC3-SCAP is retained as the metabolic-immune coupling archetype linking cholesterol biosynthesis to immune escape 73, whereas ZDHHC13-MC1R illustrates that clinically relevant palmitoylation modulation may also involve protective pro-palmitoylation rather than inhibition alone 206, 207. In parallel, PORCN and HHAT inhibitors are discussed as upstream ligand-lipidation comparators rather than as direct validation of ZDHHC-targeted therapy: WNT974 provides single-agent and combination clinical comparator data for PORCN inhibition 193, 202, ETC-159 supports genetically defined Wnt-addicted preclinical contexts 203, and RU-SKI-43/RU-SKI-201/IMP-1575 define HHAT inhibition at the chemical-probe or lead-optimization stage 194, 204, 205. Methodologically, fluorescent “turn-on” probes 208, TR-FRET-based auto-acylation readouts 51, and Acyl-Clip (209)are retained here only as discovery and validation tools for target engagement and selectivity optimization. For translational prioritization, the most pragmatic approach is therefore to rank each modality by therapeutic maturity, target-engagement feasibility, pharmacodynamic measurability, and biomarker readiness, rather than to repeat the full mechanistic cascade. The same concept of “lipidation pocket” targeting extends to transcriptional regulators. Multiple lines of TEAD-directed chemical biology support the druggability of selected lipid-binding or YAP-TEAD interface pockets: TEAD auto-palmitoylation inhibitors suppress NF2-deficient mesothelioma growth 148, TEAD lipid-pocket dysregulators induce dominant-negative Hippo-pathway inhibition 210, and newer allosteric or interface-disrupting TEAD/YAP-TEAD inhibitors show antitumor activity in defined preclinical contexts 149, 150. However, these data should be interpreted as evidence of pocket-level druggability rather than clinical validation; pharmacological blockade of TEAD-YAP also reveals context-dependent limitations, so pathway-specific biomarkers, target-engagement assays, adaptive-resistance monitoring, and tumor-context selection remain necessary before TEAD-directed strategies can be considered clinically mature 211. Finally, population- and drug-response-level evidence continues to grow. In hepatocellular carcinoma, ZDHHC5-mediated palmitoylation of CLDN4 stabilizes its lipid-raft localization, activates Notch signaling, and induces HBT lineage transdifferentiation, driving lenvatinib resistance. This phenotype can be reversed by interface-blocking compounds such as salvianolic acid B, establishing a reversible causal chain between palmitoylation, cellular phenotype, and drug response 212.

## 7. Integrative Mechanisms Across Cancers and the ZDHHC Functional Landscape: From Heterogeneity to Precision Medicine

### 7.1. Shared and Cancer-Specific Features of the ZDHHC Functional Spectrum

Building on the mechanistic sections above, this section uses recurrent ZDHHC-substrate circuits as cross-cancer stratification anchors rather than re-narrating their full pathway mechanisms. Cross-cancer comparisons reveal a conserved pro-oncogenic S-palmitoylation network centered on membrane localization, signaling coupling, and protein stabilization, which underpins malignant growth across multiple tumor types. In pancreatic ductal adenocarcinoma (PDAC), ZDHHC20 directly drives tumor proliferation and distant metastasis 127, reinforcing translational and proliferative signaling through the ZDHHC20-YTHDF3-MYC axis 213. In hepatocellular carcinoma (HCC), the ZDHHC3-SCAP-SREBP2 axis disrupts cholesterol-feedback regulation and promotes metabolic-immune crosstalk and immune evasion 73. In glioblastoma (GBM), ZDHHC9-mediated GLUT1 palmitoylation preserves plasma-membrane localization and maintains a high glycolytic state that fuels tumor growth 81. These effects also exhibit tumor-type specificity. GBM depends heavily on ZDHHC9-GLUT1 site lipidation to sustain metabolic adaptation 81; HCC relies on ZDHHC3-SCAP-SREBP2 coupling to link sterol metabolism with immune suppression 73; and PDAC is dominated by the ZDHHC20-YTHDF3-MYC pathway, which drives translation and correlates with enhanced metastatic potential 127, 213. In lung adenocarcinoma (LUAD), the ZDHHC expression profile follows a “one up, one down” dual-direction pattern: elevated ZDHHC4/12/18/24 expression associates with poor prognosis and immunosuppressive remodeling (including CD276/B7-H3 upregulation) 59, whereas ZDHHC11B downregulation suppresses EMT, indicating a context-dependent tumor-suppressive role 128. Taken together, the ZDHHC functional atlas displays both shared dependencies and cancer-specific adaptations. Universally, many tumors employ S-palmitoylation to achieve membrane anchoring and signal amplification of key receptors or substrates—such as EGFR tail palmitoylation, which fine-tunes PI3K-AKT-MYC signaling amplitude 11, 12. At the same time, each tumor type demonstrates substrate and pathway preferences: GBM emphasizes metabolic transporters 81, HCC focuses on sterol metabolism and immune coupling 73, and PDAC is centered on the translation-MYC axis and metastatic programs 127, 213. This triad—ZDHHC enzyme, substrate, and signaling pathway—thus provides a rational foundation for developing cross-cancer biomarkers and designing personalized therapeutic interventions grounded in palmitoylation biology.

### 7.2 Functional Subsets of ZDHHCs Linked to Tumor Subtypes, Immune Phenotypes, and Metastatic Propensity

At the subtype level, ZDHHC18 and ZDHHC23 define distinct glioblastoma (GBM) stem-cell niches, each sustaining different ecological states. Mechanistically, these enzymes competitively bind the E3 ligase RNF144A, thereby regulating BMI1 polyubiquitination and shaping subtype plasticity and stress adaptation within the tumor ecosystem 61. At the immune-phenotype level, ZDHHC3-centered checkpoint and metabolic-immune circuits define a lipidation-sensitive immune subset, represented by PD-L1 stabilization and DHHC3-targeted pharmacologic destabilization 27, 28, 82, as well as SCAP-dependent cholesterol remodeling and immune escape 73. DHHC9-mediated TIM-3 palmitoylation similarly marks an exhaustion-associated immune module 106. At the metastatic-regulation level, ZDHHC20 identifies a PDAC metastatic-outgrowth subset 127, whereas ZDHHC5-FAK marks an invasion/EMT module in glioblastoma 66. Collectively, these findings delineate functionally distinct ZDHHC submodules aligned with tumor subtype identity, immune phenotype, and metastatic potential—together forming a mechanistic foundation for precision stratification and therapeutic targeting across cancer contexts.

### 7.3 Functional-Atlas-Guided Personalized Therapeutic Strategies

Building on the druggable nodes already defined in Sections 4-6, the aim of this section is not to restate pathway mechanisms in full, but to translate them into a biomarker-guided framework for patient selection. Checkpoint-dominant tumors provide the clearest proof of principle: interference with PD-L1 or TIM-3 palmitoylation destabilizes inhibitory signaling and restores effector-cell activity, thereby identifying lipidation-sensitive immune phenotypes as actionable settings for therapy intensification 27, 106. A second category is represented by metabolically coupled tumors, in which ZDHHC3-SCAP-driven cholesterol rewiring in hepatocellular carcinoma and ZDHHC20-dependent stabilization of FASN define candidate contexts for combining palmitoylation-directed intervention with anti-PD-1 or metabolic therapies 68, 73. A third category comprises metastasis- and progression-dominant tumors, exemplified by ZDHHC20-dependent metastatic programs and YTHDF3-MYC signaling in pancreatic ductal adenocarcinoma, as well as EGFR-palmitoylation-dependent pathway persistence and TKI sensitivity in KRAS-mutant lung adenocarcinoma 11, 12, 127, 213. Accordingly, functional-atlas-guided personalization should integrate ZDHHC/depalmitoylase expression patterns, substrate-defined pathway dependence, and immune-metabolic phenotypes to support patient stratification, treatment prioritization, and resistance monitoring. For checkpoint lipidation blockade, the most rational approaches target PD-L1—using inhibitory peptides or small molecules—and TIM-3, via peptide-based disruption of DHHC9-mediated modification 27, 28, 106. In the metabolic-lipidation dual-target paradigm, co-inhibition of ZDHHC3-SCAP-SREBP2 together with anti-PD-1 therapy, or interference with ZDHHC20-FASN and ZDHHC20-YTHDF3-MYC axes, can simultaneously reshape metabolic flux and immune responsiveness 68, 73, 213. For tumors with high metastatic or invasive potential, selective blockade of ZDHHC20 (in PDAC metastasis) or ZDHHC5-mediated FAK palmitoylation (driving EMT and invasion in glioblastoma) represents a mechanistically grounded anti-metastatic strategy 66, 127. Finally, in LUAD, where a distinctive “one-up, one-down” expression pattern is observed—upregulation of ZDHHC4/12/18/24 correlating with poor prognosis and immunosuppressive remodeling, while ZDHHC11B downregulation confers potential tumor-suppressive effects—these molecular fingerprints provide a basis for biomarker-driven therapy design and individualized combination regimens 59, 128.

**Figure 4:**
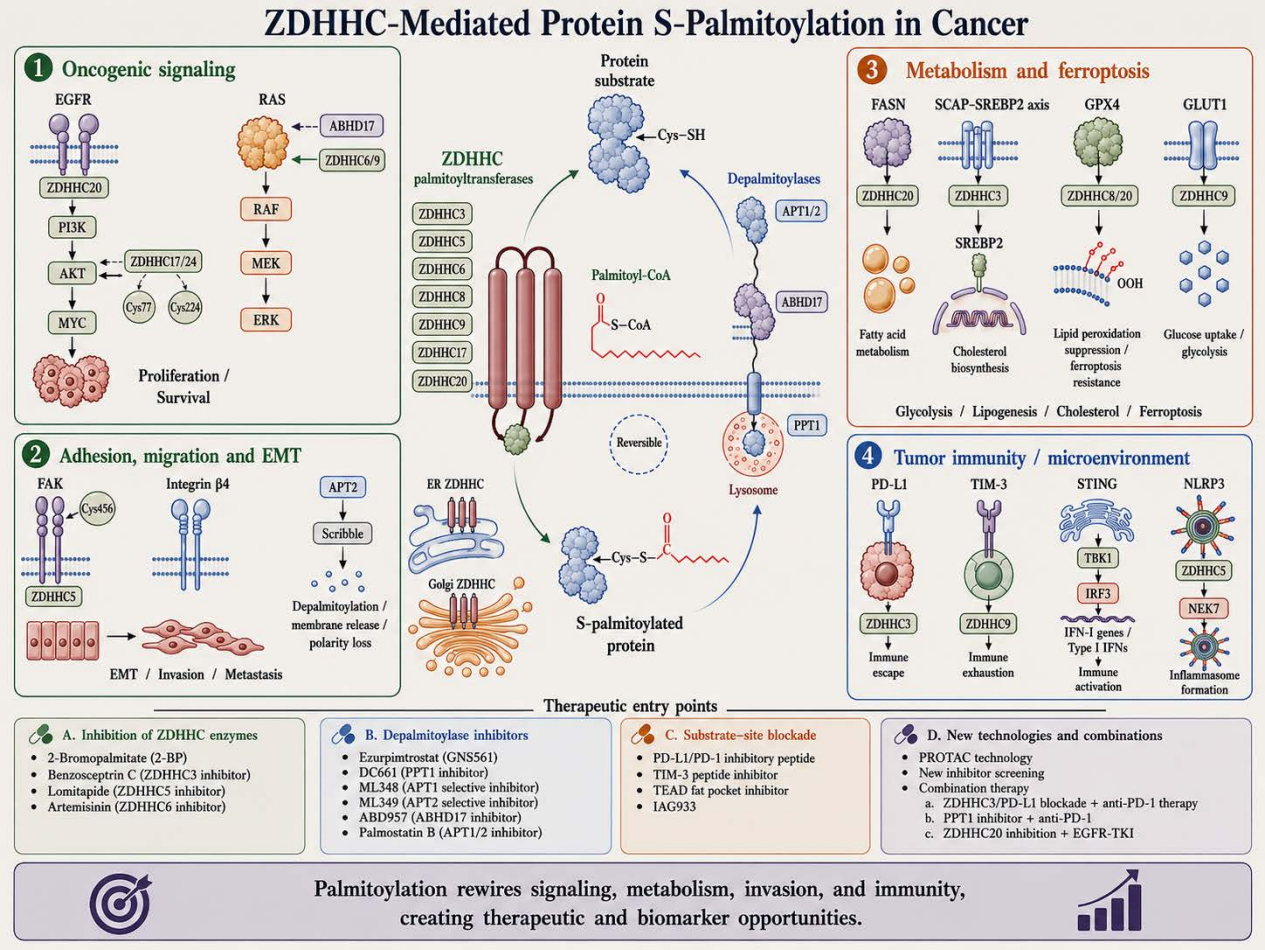
** Integrated framework of ZDHHC-mediated protein S-palmitoylation in cancer and therapeutic intervention.** The central panel illustrates the reversible ZDHHC-depalmitoylase cycle. ZDHHC palmitoyltransferases use palmitoyl-CoA as an acyl donor to catalyze thioester-linked S-palmitoylation of cysteine residues, whereas depalmitoylases, including APT1/2, ABHD17 family members, and PPT1, remove acyl groups to dynamically regulate substrate membrane residency, subcellular trafficking, and signaling output. The surrounding modules summarize representative cancer-relevant circuits. In oncogenic signaling, ZDHHC20-mediated EGFR palmitoylation, ZDHHC17/24-mediated AKT modification at Cys77/Cys224, and ZDHHC6/9-regulated RAS palmitoylation support PI3K-AKT-MYC and RAS-RAF-MEK-ERK pathway output. In adhesion, migration, and EMT, ZDHHC5-dependent FAK palmitoylation at Cys456 promotes membrane localization and invasive signaling, whereas APT2-mediated Scribble depalmitoylation contributes to membrane release and polarity loss. In metabolism and ferroptosis, ZDHHC-dependent regulation of GLUT1, FASN, SCAP-SREBP2, and GPX4 links glycolytic flux, fatty-acid metabolism, cholesterol biosynthesis, ferroptosis resistance, and therapeutic vulnerability. In tumor immunity and the microenvironment, palmitoylation stabilizes immune-checkpoint proteins such as PD-L1 and TIM-3 and regulates innate immune nodes including STING and NLRP3, thereby shaping immune escape, immune exhaustion, type I interferon signaling, and inflammasome activation. The lower panels summarize therapeutic entry points, including ZDHHC enzyme inhibition, depalmitoylase blockade, substrate-site intervention, and emerging technology- or combination-based strategies with immune-checkpoint or targeted therapies. EMT, epithelial-mesenchymal transition; IFN-I, type I interferon; TKI, tyrosine kinase inhibitor.

Together, these observations support a circuit-level model in which ZDHHC enzymes, depalmitoylases, substrate-defined dependencies, and tumor context jointly shape oncogenic signaling, invasion, metabolism, ferroptotic vulnerability, and immune phenotype 11, 12, 66, 73, 81, 83, 84, 106, 127, 213. This integrated framework links the reversible palmitoylation-depalmitoylation cycle to representative cancer-relevant modules and therapeutic entry points, providing a visual summary of how palmitoylation biology can inform pathway prioritization, combination design, and biomarker-guided translation 197, 198** (Figure 4)**.

## 8. Future Perspectives: From Fundamental Mechanisms to Precision Translation

As discussed in Section 5, depalmitoylases may exert non-catalytic architectural functions in addition to their canonical catalytic roles 188. In this Future Perspectives section, we therefore do not reintroduce these findings as a separate mechanistic subsection, but instead highlight the unresolved questions they raise: how broadly APT1/2 scaffold-like functions operate across tumor contexts, whether catalytic and non-catalytic dependencies can be separated pharmacologically, and how nuclear-proximal lipidation events can be mapped with site- and localization-resolved tools. Examples such as APT1/2-RAD21/BRG1 interactions, APT1/nuclear-EGFR signaling in osimertinib resistance, and nuclear PORCN-mediated Ku70 palmitoylation are retained here only as future-mapping priorities rather than as a repeated mechanistic narrative 188, 214, 215. Emerging tools such as SwissKASH and proximity-labeling proteomics may help define which nuclear or scaffold-like lipidation events are recurrent, disease-relevant, and therapeutically separable from canonical palmitoylation-cycle regulation 216, 217.

### 8.1 Multi-Omics Databases and Visualization Platforms for Palmitoylation Research

A comprehensive understanding of the ZDHHC-substrate network requires a unified and reliable data infrastructure. The SwissPalm database serves as a foundational resource, integrating cross-species and cross-cell-type palmitoyl-proteome datasets, anchored by site-level experimental evidence that enables standardized search and comparison 218. Extending this framework, CellPalmSeq provides a visualized and downloadable transcriptomic atlas of palmitoyltransferases and depalmitoylases across human cell types, tissues, and tumor cell lines—allowing researchers to explore regulatory patterns across single-cell, tissue, and cancer hierarchies 219. In the context of the nervous system, BrainPalmSeq maps the spatial expression, substrate enrichment, and functional annotation of palmitoylation enzymes across mouse brain regions and cell populations, suggesting that distinct spatial expression patterns of ZDHHCs may predict their substrate and pathway specificity 220. For in situ visualization and functional readouts, the SwissKASH system enables site- and localization-specific detection of S-palmitoylation within living cells, providing a practical imaging framework to address the question of “where and by which enzyme” a substrate is modified 216. Complementing this, integrative “omics × proximity-labeling” approaches merge palmitoyl-proteomic data with the APT1/2 interaction landscape, thereby defining pathway-level dependencies and facilitating mechanistic tracking and candidate prioritization within disease models 217. At the algorithmic and interactive exploration level, the TopoPalmTree (2025) platform employs a gradient-boosting model trained on multi-species experimental data with interpretable feature sets, offering high-confidence predictions of near-membrane palmitoylation sites in transmembrane proteins. This framework closes the loop from database to hypothesis to experimental validation, streamlining data-driven discovery 221. To ensure data reproducibility, reusability, and large-cohort integration, palmitoyl-proteomic datasets and their raw mass spectrometry files should be archived under the ProteomeXchange/PRIDE standards, leveraging their visualization and re-analysis pipelines to establish a standardized workflow from raw spectra to curated protein maps 222.

### 8.2 Preclinical Validation and Combination Strategies for Selective ZDHHC Inhibitors

The dual advancement of chemical tools and structural biology is rapidly transforming the discovery pipeline from mechanistic insight to lead-compound generation. High-throughput screening (HTS) platforms based on TR-FRET auto-acylation readouts and acyl-cLIP assays for ZDHHC3/7/20 have now been established, enabling robust identification of S-acylation inhibitors in both cell-free and cellular systems 34, 51. At the rational-design level, the “hydrophobic cavity-dual-site recognition” architecture of human ZDHHC20 in complex with acyl-CoA has been resolved, providing a direct structural template for binding-pocket definition, ligand anchoring, and structure-activity optimization 33. From a pharmacologic perspective, new strategies are emerging that combine enzyme selectivity with immunologic synergy. The marine natural product Benzosceptrin C has been shown to inhibit DHHC3, block PD-L1 palmitoylation, and promote its lysosomal degradation, thereby amplifying anti-tumor immunity when co-administered with CTLA-4 blockade in mouse models—an illustrative proof of concept for enzyme-checkpoint co-targeting 28. Parallel efforts have produced a targeted-protein-degradation (TPD) approach against ICB-resistant tumors: cell-penetrating peptide-induced chimeric conjugates (cp-PCCs) that selectively degrade DHHC3, reduce PD-L1 lipidation, and restore immune responsiveness in resistant models, establishing a methodological prototype for enzyme-directed TPD development 191. Looking ahead, preclinical optimization should integrate structure-activity relationships (SAR) with substrate-selectivity profiling to balance isoform specificity and pharmacologic safety windows. A longitudinal evaluation linking pharmacodynamics, pharmacokinetics, and immunodynamics under combination settings—particularly with immune checkpoint blockade (ICB) or kinase inhibition—will be essential. Concurrently, employing orthogonal HTS and secondary-validation systems—including TR-FRET, acyl-cLIP, and fluorescent “turn-on” probes—can minimize false positives and significantly shorten the iteration cycle from initial hit to validated lead compound 208.

### 8.3 Clinical Biomarker and Companion-Diagnostic Development: Candidate Markers, Detection Strategies, and Validation Requirements

Clinical translation of palmitoylation biology requires separating candidate biomarkers into three non-equivalent layers: enzyme-expression or enzyme-activity biomarkers, substrate-palmitoylation biomarkers, and pathway-level pharmacodynamic biomarkers 197, 198. Enzyme-expression biomarkers are currently the most accessible in clinical specimens. Examples include ZDHHC4/12/18/24 and APT2 expression patterns associated with prognosis and immune infiltration in lung adenocarcinoma 59, reduced ZDHHC11B expression as a tumor-suppressive feature in lung adenocarcinoma 128, ZDHHC2-linked AGK palmitoylation and sunitinib resistance in renal carcinoma 77, and PPT1-positive macrophage states associated with an immunosuppressive contexture and immunotherapy responsiveness in hepatocellular carcinoma 38. However, expression alone should be interpreted as a prognostic, resistance-associated, or microenvironmental-context marker rather than as proof of palmitoylation-circuit dependence 197.

Substrate-palmitoylation biomarkers are mechanistically closer to drug response but are more difficult to implement clinically. The most compelling candidates include checkpoint substrates such as PD-1 25, PD-L1 27, 28, 82, TIM-3 106, and B7-H4 154; metabolic-immune nodes such as SCAP 73 and FASN 68; ferroptosis regulators such as GPX4 83, 84; and resistance-associated substrates such as CLDN4 212 and YTHDF3 213. These markers are most informative when substrate lipidation is linked to a defined writing or erasing enzyme, site-level modification, pathway output, and treatment response 29, 35, 40, 197. For example, ZDHHC3-SCAP may define cholesterol-driven immune-evasive HCC contexts 73, ZDHHC20-FASN and ZDHHC20-YTHDF3 may identify metabolic or translational dependencies 68, 213, ZDHHC8/20-GPX4 may inform ferroptosis sensitivity 83, 84, and ZDHHC5-CLDN4 may support resistance monitoring during lenvatinib treatment 212.

Detection methodology remains the main barrier to companion-diagnostic development. ZDHHC or depalmitoylase expression can be measured by RNA sequencing, quantitative PCR, immunohistochemistry, multiplex immunofluorescence, or spatial transcriptomic/proteomic approaches, but these assays do not directly quantify substrate palmitoylation 59, 197, 219. By contrast, acyl-biotin exchange, acyl-resin-assisted capture, click-chemistry profiling, targeted mass spectrometry, and ZDHHC-specific chemical-genetic mapping can detect palmitoylated substrates, but they usually require optimized tissue handling, sufficient protein input, preserved thioester chemistry, and careful normalization for substrate abundance and subcellular compartmentalization 29, 35, 40. Clinical implementation will therefore require paired measurement of total substrate abundance and site- or enzyme-linked palmitoylation status, ideally in fresh-frozen or prospectively collected biopsy material 197, 198. Current databases and infrastructures, including SwissPalm, CellPalmSeq, and ProteomeXchange/PRIDE-compatible deposition of palmitoyl-proteomic datasets, can support assay harmonization, cross-cohort comparison, and reproducibility assessment 218, 219, 222.

Liquid-biopsy development is even less mature. Circulating tumor DNA, cell-free RNA, extracellular vesicles, or plasma proteins may eventually provide indirect read-outs of ZDHHC expression, depalmitoylase activity, or substrate abundance, but direct quantification of thioester-linked S-palmitoylation occupancy in blood-based specimens is not yet clinically validated 223. Therefore, liquid-biopsy approaches should currently be regarded as exploratory and should be anchored to paired tumor-biopsy validation before being used for patient selection 197, 223.

A practical validation framework should proceed in four steps 197, 198. First, discovery studies should identify candidate ZDHHC/depalmitoylase expression patterns or substrate-palmitoylation events in well-annotated tumor cohorts. Second, analytical validation should establish assay specificity, sensitivity, reproducibility, dynamic range, pre-analytical stability, and orthogonal confirmation of the lipidated substrate. Third, clinical validation should test whether the biomarker predicts diagnosis, prognosis, pharmacodynamic target engagement, or response to a defined therapy in independent cohorts. Fourth, clinical utility should be assessed prospectively in biomarker-enriched or biomarker-stratified trials, ideally with predefined cutoffs and locked assays. Under this framework, current palmitoylation-related biomarkers remain promising but exploratory; none yet qualifies as a clinically validated companion diagnostic for palmitoylation-directed therapy 197, 198.

### 8.4 Extension of Palmitoylation Beyond Oncology and Cross-Disease Therapeutic Applications

Emerging evidence indicates that the regulatory principles of protein palmitoylation extend well beyond oncology, encompassing the immune, metabolic, and nervous systems, where analogous lipidation switches define signaling plasticity and therapeutic vulnerability. In immune regulation, the inhibitory receptor TIM-3 exemplifies a transferable lipidation target. ZDHHC9-catalyzed palmitoylation of Cys^296 prevents HRD1-mediated ubiquitination and degradation, stabilizing TIM-3 at the plasma membrane and driving T/NK-cell exhaustion. A rationally designed blocking peptide that interferes with this site-specific modification restores CAR-T and NK-cell cytotoxicity, enhancing antitumor immunity in vivo 106. Similarly, across tumor contexts, independent studies have established that PD-L1 palmitoylation directly governs its stability and surface retention. The marine natural product Benzosceptrin C selectively inhibits DHHC3, inducing PD-L1 lysosomal degradation and synergizing with anti-PD-1/PD-L1 therapy 28. Complementary chemical-biologic approaches have yielded cell-penetrating peptide-induced chimeric conjugates (cp-PCCs) that promote selective degradation of DHHC3, suppress PD-L1 lipidation, and overcome immune-checkpoint blockade (ICB) resistance, while early human and animal data demonstrate that competitive peptides targeting the PD-L1 palmitoylation site can augment T-cell cytotoxicity and potentiate ICB efficacy 27, 191. At the intersection of innate immunity and vascular biology, STING activation depends critically on palmitoylation at Cys^91, which facilitates its interaction with JAK1 and the downstream IFN-I-JAK1-STAT1 axis, thereby promoting vascular normalization and CD8⁺ T-cell infiltration—a mechanism directly coupled to antitumor immunity 156. Single-molecule imaging further reveals that STING clustering at the trans-Golgi network requires both palmitoylation and local cholesterol enrichment, providing a structural basis for signal amplification and spatial organization 158. Notably, pulsed-electric-field stimulation can induce STING palmitoylation and oligomerization in skeletal muscle, offering an engineerable route to modulate inflammation and infection-related immunity 224. In metabolic inflammation, palmitoylation has emerged as a key driver of nonalcoholic steatohepatitis (NASH). Under metabolic stress, ZDHHC3 expression is upregulated, leading to site-specific palmitoylation of the inflammatory modulator IRHOM2, which anchors it to membranes and prevents K48-linked ubiquitination and degradation, thereby exacerbating hepatic inflammation and fibrosis. Conversely, ZDHHC3 knockout or inhibition significantly alleviates NASH progression in vivo 225. Within the nervous system, palmitoylation defects contribute to neurodegeneration. In Huntington's disease models, reduced palmitoylation of the selective substrate SQSTM1/p62 disrupts autophagy and accelerates neuronal pathology, whereas restoration of this modification ameliorates both cellular and organismal phenotypes, underscoring a functional “lipidation-autophagy-neurodegeneration” axis with translational potential 226. Broader cross-context evidence further supports the generality of this mechanism. In lung adenocarcinoma and pancreatic cancer, ZDHHC9 promotes immune suppression and resistance to ICB by stabilizing PD-L1 palmitoylation and remodeling the tumor microenvironment; conversely, genetic or pharmacologic inhibition of ZDHHC9 reduces PD-L1 expression and enhances the efficacy of anti-PD-L1 therapy, establishing an enzyme-checkpoint co-targeting paradigm with cross-disease relevance 72, 227. From a systems perspective, recent integrative reviews have positioned palmitoylation as a shared regulatory axis spanning the cancer-cardiometabolic-immune-neural continuum, emphasizing the need for translational frameworks that combine multi-omics mapping, selective inhibitors, and patient stratification to enable cross-disciplinary therapeutic evaluation and precision application 165.

## 9. Conclusions

ZDHHC-mediated protein S-palmitoylation has emerged as a central regulatory layer that links membrane topology to oncogenic signaling, metabolic and ferroptotic thresholds, chromatin organisation and immune phenotypes across diverse cancers. At the biochemical level, recent structural studies of human ZDHHC enzymes and their cofactors now connect acyl-pocket geometry and ankyrin-repeat-mediated substrate capture to site and chain-length selectivity. Functionally, multi-omics analyses and disease models converge on a relatively small set of palmitoylation-sensitive hubs—including EGFR-AKT-RAS, FAK-EMT, SCAP/SREBP, GPX4/SLC7A11, PD-L1/TIM-3, cGAS-STING and NLRP3—that collectively shape tumor growth, metastatic capacity and immune escape.

Crucially, several ZDHHC-substrate circuits directly couple lipidation to epigenetic regulation and DNA repair. ZDHHC16-dependent SETD2 palmitoylation affects H3K36me3 deposition and radioresistance; ZDHHC1-mediated p53 lipidation interfaces with DNMT3A-driven promoter methylation; ZDHHC5-EZH2 coupling supports stemness programmes in p53-mutant glioma; and nuclear PORCN-driven Ku70 palmitoylation promotes non-homologous end-joining. In parallel, non-enzymatic scaffolding functions of depalmitoylases such as APT1/2—bridging BRG1 and RAD21—have revealed a nuclear dimension of the palmitoylation network that impacts chromatin topology and stress tolerance. Together, these findings position S-palmitoylation as a bona fide epigenetic interface rather than a peripheral modifier of signaling proteins.

Translationally, the palmitoylation-depalmitoylation cycle is now being interrogated at multiple levels. Among depalmitoylase-directed strategies, PPT1-directed agents illustrate both the progress and the current limits of this field: GNS561/Ezurpimtrostat has first-in-human evidence of safety, hepatic exposure, and disease stabilization, but no established objective-response efficacy or validated predictive biomarker 24, whereas DC661 remains preclinical and supports PPT1 targeting through chloroquine-derivative biology and hepatocellular-carcinoma sorafenib-sensitization models 23, 196. Mechanistic studies support lysosomal/autophagy modulation by GNS561 in hepatocellular carcinoma models 26, and immune-context modulation has been suggested by PPT1-positive macrophage states in HCC (38)and by anti-PD-1 combination studies in melanoma and HCC models 169, 170; however, durable clinical benefit, substrate-level target engagement, optimal patient selection, and biomarker-linked validation remain unresolved 24. Other intervention classes—including ABHD17 inhibition 50, 94, immune-checkpoint palmitoylation blockade involving PD-1, PD-L1, TIM-3, and B7-H4 25, 27, 28, 106, 154, and upstream PORCN/HHAT ligand-lipidation blockade 193, 194—are mechanistically grounded but should still be considered clinically immature strategies requiring prospective pharmacodynamic and biomarker-linked validation. At the same time, experience with broad-spectrum tools such as 2-bromopalmitate and Palmostatin B underscores the need for a stringent three-tier evidence chain—site, enzyme and pathway—to ensure mechanistic fidelity.

Looking forward, several priorities are clear. First, systematic palmitoyl-proteomics integrated with single-cell transcriptomics is needed to build “ZDHHC-substrate-phenotype” atlases that resolve tissue- and subtype-specific dependencies, particularly where palmitoylation intersects with chromatin regulation and DNA repair. Second, high-throughput structural and chemical-biology platforms—including TR-FRET, acyl-cLIP, fluorescent “turn-on” assays and topology-aware prediction algorithms—should be leveraged to accelerate discovery of isoform-selective DHHC and depalmitoylase inhibitors with acceptable pharmacokinetics and safety. Third, palmitoylation fingerprints and ZDHHC/depalmitoylase expression patterns merit evaluation as biomarkers for patient stratification, resistance monitoring and pharmacodynamic read-outs in early-phase trials.

Ultimately, palmitoylation-directed interventions are likely to be most effective as components of rational combination regimens rather than stand-alone therapies. Enzyme- and site-selective modulation of S-palmitoylation, particularly when integrated with immune-checkpoint inhibitors, kinase inhibitors, or metabolic agents, can become clinically meaningful in precision oncology only if three conditions are demonstrated prospectively: selective and tolerable target modulation, substrate- or pathway-level pharmacodynamic confirmation in patient specimens, and biomarker-guided enrichment of tumor contexts that are demonstrably dependent on the relevant palmitoylation circuit.

## Figures and Tables

**Table 1:** ZDHHC family in cancer: structural, mechanistic, and phenotypic landscape.

Enzyme	Localization / Partner Complex	Representative Substrate (site)	Principal Pathway / Function	Cancer Model → Phenotype	Evidence tier and basis (Ref.)
ZDHHC20	Golgi / ER; stabilized by GOLGA7b	EGFR (C-terminal) (11, 12); FASN (C1471/C1881) (68); GPX4 (C66) (84)	PI3K-AKT-MYC axis; lipogenesis; ferroptosis resistance	NSCLC: EGFR palmitoylation sustains TKI dependence (11, 12); HCC: FASN stabilization drives tumorigenesis (68)	Structural biology + cell + in vivo (11, 12, 32-34, 41, 42, 68, 84)
ZDHHC3 (DHHC3)	Golgi network	SCAP (S-acyl) (73); PD-L1 (cytosolic tail) (27, 28, 82)	Cholesterol biosynthesis / immune checkpoint stabilization	HCC: SCAP S-acylation activates SREBP & immune escape (73); PD-L1 stabilization suppresses T-cell cytotoxicity (27, 28)	Cell + animal validation (27, 28, 73, 82)
ZDHHC9	Golgi / plasma membrane	GLUT1 (C207) (81); LDHA (C163) (71); TIM-3 (C296) (106)	Glycolysis; lactate flux; T/NK exhaustion	GBM: ↑ glycolytic flux (81); PDAC: chemoresistance via LDHA lipidation (71); TIM-3 stabilization → immune exhaustion (106)	Robust mechanistic & in vivo evidence (71, 72, 81, 106)
ZDHHC5	Plasma membrane; anchored by GOLGA7b	FAK (C456) (66); EZH2 (64)	Adhesion / EMT / stemness programs	GBM invasion ↑ (66); p53-mutant glioma EZH2 axis activation (64)	Medium (64, 66)
ZDHHC6	ER; complex with SELENOK (48, 228)	PPARγ (69); IP3R (48)	Lipid metabolism; Ca²⁺ signaling	CRC: PPARγ stabilization → lipogenic reprogramming (69)	Medium (48, 69)
ZDHHC4	ER / mitochondrial interface (CPT1A-dependent recruitment) (155)	GSK3β (C14) (62); MAVS (C79) (155)	EZH2-STAT3 axis; IFN-I amplification	GBM: TMZ resistance ↑ (62); epigenetic stress → IFN-I ↑ (155)	Medium (155, 62)
ZDHHC7	Golgi / PM	ATG16L1 (C153) (102); Scribble (123); AR axis (74)	Autophagy; cell polarity; androgen signaling	Prostate cancer AR inhibition (74); OCCC: YAP-linked ferroptosis resistance (144)	Medium (74, 102, 123, 144)
ZDHHC11B	Cytoplasm / PM	—	EMT suppression / tumor restraint	LUAD down-regulated → EMT inhibition (128)	Medium (128)

**Abbreviations:** NSCLC, non-small-cell lung cancer; HCC, hepatocellular carcinoma; GBM, glioblastoma; PDAC, pancreatic ductal adenocarcinoma; LUAD, lung adenocarcinoma; OCCC, ovarian clear cell carcinoma; TKI, tyrosine kinase inhibitor; IFN-I, type-I interferon; EMT, epithelial-mesenchymal transition. Evidence tiers are defined as follows: Tier 1, convergent site-level, enzyme-level, biochemical, functional, and in vivo validation; Tier 2, mechanistic support in limited model systems or incomplete rescue settings; Tier 3, primarily correlative, expression-based, pharmacological, or hypothesis-generating evidence.

**Table 2:** Palmitoylation-driven mechanistic circuits linking molecular sites to oncogenic phenotypes.

Substrate (site)	Writing enzyme / Depalmitoylase	Structural / Localization Effect	Downstream Pathway Output	Biological Phenotype	Therapeutic Leverage (Ref.)
EGFR (C-tail)	ZDHHC20 (11, 12)	Stabilizes PI3K complex; enforces signal amplitude	PI3K-AKT-MYC	NSCLC growth & TKI dependence	DHHC20 inhibition ↑ TKI sensitivity (11, 12)
PD-L1 (cytosolic tail)	ZDHHC3 (27, 28, 82)	Prevents ubiquitination/lysosomal degradation; membrane stabilization	Immune checkpoint maintenance	Immune evasion ↑	DHHC3 inhibitor (Benzosceptrin C) + ICB synergy (28)
AKT (C77/C224)	ZDHHC17/24 (91); APT2 (49)	Enables PIP3-independent membrane anchoring	AKT persistent activation	NASH → HCC progression	Cell-penetrating peptide blocks palmitoylation → ↓ tumor growth (91)
GLUT1 (C207)	ZDHHC9 (81)	Sustains plasma-membrane residency	Glycolytic flux ↑	GBM growth ↑	DHHC9 blockade → ↓ glycolysis & growth (81)
LDHA (C163)	ZDHHC9 (71)	Alters tetramer assembly & activity	Metabolic reprogramming	PDAC chemoresistance ↑	DHHC9 inhibition + chemotherapy synergy (71, 72)
GPX4 (C66/C75)	ZDHHC20 (84); ZDHHC8 (83); APT2 (49)	Increases GPX4 stability; raises ferroptosis threshold	ROS/lipid peroxidation control	Ferroptosis resistance ↑	Inhibit ZDHHC8/20 → ICB sensitization (83, 84)
TIM-3 (C296)	ZDHHC9 (106)	Evades HRD1-mediated ubiquitination; membrane stabilization	Exhaustion program ↑	Immune escape	Blocking peptide + CAR-T/NK enhancement (106)
SCAP (S-acyl)	ZDHHC3 (73)	Disrupts cholesterol feedback loop	SREBP1/2 activation	HCC immune escape	DHHC3 inhibition + PD-1 synergy (73)
FAK (C456)	ZDHHC5 (66)	Promotes PM anchoring & kinase activation	EMT / migration	GBM invasion ↑	ZDHHC5 knockdown → ↓ EMT (66)
GSK3β (C14)	ZDHHC4 (62)	Strengthens EZH2-STAT3 axis	Stemness / drug resistance	GBM TMZ resistance ↑	ZDHHC4 inhibition sensitizes to TMZ (62)

**Abbreviations:** NSCLC, non-small-cell lung cancer; HCC, hepatocellular carcinoma; GBM, glioblastoma; PDAC, pancreatic ductal adenocarcinoma; ICB, immune checkpoint blockade; PM, plasma membrane; PIP3, phosphatidylinositol-(3,4,5)-trisphosphate; ROS, reactive oxygen species; TMZ, temozolomide; EMT, epithelial-mesenchymal transition.

**Table 3:** Therapeutic interventions targeting the palmitoylation axis and translational opportunities.

Agent / Modality	Primary Target (Axis)	Mechanism of Action / Selectivity Note	Key Pre-clinical / Clinical Evidence	Combination Window	Development Stage	Evidence tier	Ref.
GNS561 (Ezurpimtrostat)	PPT1 (depalmitoylase)	Oral, lysosomotropic PPT1 inhibitor; deacidifies lysosomes → late-stage autophagy blockade; strong liver exposure	Preclinical HCC activity via lysosomal modulation; Phase I safety/hepatic exposure with stable disease; no established objective response or predictive biomarker	+ anti-PD-1: CD8⁺ repopulation/infiltration rationale; ± TKI/chemo: exploratory	Early clinical (Phase I completed)	A	(24, 26, 169, 170)
DC661	PPT1	Dimeric chloroquine analogue; acid-stable direct PPT1 inhibitor; autophagy and lysosomal lipid-peroxidation-linked cell death	Identified as CQ-class PPT1 target; sensitizes HCC to sorafenib; PPT1 inhibition augments anti-PD-1 activity in melanoma models	+ anti-PD-1; + sorafenib (HCC)	Pre-clinical	B	(23, 169, 196)
ABD957	ABHD17 family (depalmitoylases)	Selective covalent ABHD17A/B/C inhibitor; impairs N-Ras depalmitoylation	NRAS-dependent models: MAPK signaling attenuation; synergy with MEK inhibitors demonstrated	+ MEK inhibitors	Lead / pre-clinical	B	(50, 94)
Benzosceptrin C	DHHC3	Marine natural product; inhibits DHHC3 → prevents PD-L1 palmitoylation → lysosomal degradation; lowers PD-1 binding	In vivo (MC38): enhanced T-cell cytotoxicity; effective with anti-CTLA-4; mechanistic link to PD-L1 palmitoylation established	+ anti-CTLA-4 (shown); ± anti-PD-1/PD-L1 (rationale)	Lead / pre-clinical	B	(27, 28)
WNT974 (LGK974)	PORCN (Wnt O-acylation)	First-in-class PORCN inhibitor; blocks Wnt O-acylation (palmitoleoylation) and secretion	Phase I single-agent completed; Phase Ib/II combo with encorafenib + cetuximab in BRAF V600E mCRC explored	+ BRAF/EGFR-pathway drugs	Phase I; Phase Ib/II (combo)	A	(193, 202)
RU-SKI-43 / IMP-1575	HHAT (Shh N-palmitoylation)	RU-SKI-43 inhibits HHAT (selectivity caveats; off-target cytotoxicity reported); IMP-1575 is a defined-site, potent HHAT tool inhibitor (binding site resolved)	Shh-driven signaling inhibition (pre-clinical); structural/biophysical definition of IMP-1575-HHAT binding	± SMO inhibitors (mechanistic rationale; not yet clinically validated)	Chemical probes / lead	B	(194, 204, 205)
cp-PCC chimeras (e.g., PCC-16)	DHHC3 (TPD)	Cell-penetrating peptide-induced chimeras that degrade DHHC3 → PD-L1 down-regulation; targeted protein degradation approach	In ICB-resistant models: restored antitumor immunity; formal correction published	+ ICB	Pre-clinical	B	(191)
Lomitapide (repurposed)	ZDHHC5	Approved lipid-lowering agent repositioned to block ZDHHC5-dependent SSTR5 palmitoylation	PDAC pre-clinical models: anti-proliferative efficacy with mechanistic closure	+ chemo / targeted agents (rationale)	Pre-clinical	B	(201)
2-Bromopalmitate / Palmostatin-B *(tools)*	Broad PAT / APT inhibition	2-BP: non-selective palmitoylation blocker with multiple off-targets (incl. APT1/2; lipid enzymes); Palm-B: APT1/2 inhibitor disrupting Ras depalmitoylation	Mechanistic probes only; not suitable for selectivity conclusions	—	Tool compounds	C	(21, 22, 53, 54)

**Abbreviations**: APT, acyl-protein thioesterase; ICB, immune-checkpoint blockade; PAT, palmitoyl acyltransferase; PDAC, pancreatic ductal adenocarcinoma; SMO, Smoothened; TKI, tyrosine-kinase inhibitor; TPD, targeted protein degradation. Evidence tiers denote translational maturity rather than proven clinical efficacy. Tier A indicates clinical-stage or human-exposure evidence, including safety, pharmacokinetic, or early disease-control data, without necessarily establishing objective efficacy or validated predictive biomarkers. Tier B indicates mechanistically supported preclinical or lead-stage evidence with in vivo validation but no definitive human efficacy data. Tier C indicates tool-compound or hypothesis-generating evidence, useful for mechanistic interrogation but insufficient for selectivity or therapeutic-efficacy claims.

## Abbreviations

ABHD: α/β-Hydrolase domain-containing protein
ACLY: ATP-citrate lyase
ACOX1: Acyl-CoA oxidase 1
AGK: Acylglycerol kinase
AKT: Protein kinase B
ALDH: Aldehyde dehydrogenase
AML: Acute myeloid leukemia
AMPK: AMP-activated protein kinase
AR: Androgen receptor
ATG: Autophagy-related protein
BAX: BCL2-associated X protein
BRD4: Bromodomain-containing protein 4
BRG1: Brahma-related gene 1 (SMARCA4)
CAF: Cancer-associated fibroblast
CAR-T: Chimeric antigen receptor T cell
ccRCC: Clear-cell renal cell carcinoma
CD36: Cluster of differentiation 36
CD82/KAI1: Cluster of differentiation 82 / Kang-ai 1
CDK: Cyclin-dependent kinase
CRD: Cysteine-rich domain
CRC: Colorectal cancer
CTLA-4: Cytotoxic T-lymphocyte-associated protein 4
DC661: Dimeric chloroquine 661 (PPT1 inhibitor)
DHHC/ZDHHC: Asp-His-His-Cys motif-containing palmitoyl acyltransferase
DNMT3A: DNA methyltransferase 3A
EGFR: Epidermal growth factor receptor
EMT: Epithelial-mesenchymal transition
ER: Endoplasmic reticulum
ERK: Extracellular signal-regulated kinase
ESCC: Esophageal squamous cell carcinoma
EZH2: Enhancer of zeste homologue 2
FAK: Focal adhesion kinase
FASN: Fatty acid synthase
FZD: Frizzled receptor
GBM: Glioblastoma
GLUT1: Glucose transporter 1
GPX4: Glutathione peroxidase 4
GSC: Glioma stem cell
GSDMD: Gasdermin D
GSDME: Gasdermin E
HAT (HHAT): Hedgehog acyltransferase
HBT: Hepatic-to-biliary lineage transition
HCC: Hepatocellular carcinoma
HIF-1α: Hypoxia-inducible factor-1α
HHAT: Hedgehog acyltransferase
ICB: Immune-checkpoint blockade
IFN-I: Type I interferon
IRHOM2: Rhomboid-like protein 2 (iRhom2)
JAK: Janus kinase
KIRC: Kidney renal clear-cell carcinoma
LDHA: Lactate dehydrogenase A
lncRNA: Long non-coding RNA
LUAD: Lung adenocarcinoma
MAPK: Mitogen-activated protein kinase
MC1R: Melanocortin-1 receptor
MDH2: Mitochondrial malate dehydrogenase 2
MBOAT: Membrane-bound O-acyltransferase
mCRC: Metastatic colorectal cancer
mTOR: Mechanistic target of rapamycin
NASH: Non-alcoholic steatohepatitis
NF-κB: Nuclear factor-κB
NLRP3: NOD-like receptor family pyrin domain-containing 3
NSCLC: Non-small-cell lung cancer
OCCC: Ovarian clear-cell carcinoma
PD-1: Programmed cell death protein 1
PD-L1: Programmed death-ligand 1
PDAC: Pancreatic ductal adenocarcinoma
PHF2: Plant homeodomain finger protein 2
PORCN: Porcupine O-acyltransferase
PRR: Pattern-recognition receptor
PPT1: Palmitoyl-protein thioesterase 1
PTM: Post-translational modification
RAS: Rat sarcoma viral oncogene homologue
ROS: Reactive oxygen species
SCAP: SREBP cleavage-activating protein
SETD2: SET domain containing 2
SLC7A11: Solute carrier family 7 member 11
SMO: Smoothened receptor
SREBP: Sterol regulatory element-binding protein
STAT3: Signal transducer and activator of transcription 3
STING: Stimulator of interferon genes
TAZ: Transcriptional co-activator with PDZ-binding motif
TEAD: TEA domain transcription factor
TGN: Trans-Golgi network
TIM-3: T-cell immunoglobulin and mucin-domain containing-3
TKI: Tyrosine-kinase inhibitor
TMZ: Temozolomide
TNBC: Triple-negative breast cancer
TPD: Targeted protein degradation
VEGF: Vascular endothelial growth factor
YAP: Yes-associated protein
